# Three Spirometry‐Based At‐Risk Phenotypes Predict Incident Airflow Limitation: A Population‐Based Study

**DOI:** 10.1002/resp.70296

**Published:** 2026-08-11

**Authors:** Hendrik Pott, Roman Martin, Wilhelm Bertrams, Mustafa Abdo, Margit Heier, Annette Peters, Beate Stubbe, Max Kutzinski, Anne Obst, Ralf Ewert, Dominik Heider, Henry Völzke, Gernot Rohde, Claus F. Vogelmeier, Bernd Schmeck, Stefan Karrasch, the KORA‐study group

**Affiliations:** ^1^ Department of Medicine, Pulmonary and Critical Care Medicine Clinic for Airway Infections, University Medical Centre Marburg, Philipps‐University Marburg Marburg Germany; ^2^ Institute for Lung Research, Universities of Giessen and Marburg Lung Centre, Philipps‐University Marburg Marburg Germany; ^3^ Institute of Medical Informatics, University of Münster Münster Germany; ^4^ LungenClinic Grosshansdorf and Airway Research Center North Grosshansdorf Germany; ^5^ Institute of Epidemiology, Helmholtz Zentrum München ‐ German Research Center for Environmental Health (GmbH) Neuherberg Germany; ^6^ KORA Study Centre, University Hospital Augsburg Augsburg Germany; ^7^ Institute for Medical Information Processing, Biometry and Epidemiology (IBE), Faculty of Medicine, LMU Munich Munich Germany; ^8^ German Centre for Cardiovascular Research (DZHK), Partner Site: Munich Heart Alliance Munich Germany; ^9^ Institute and Clinic for Occupational, Social and Environmental Medicine, LMU University Hospital, LMU Munich Munich Germany; ^10^ Comprehensive Pneumology Center Munich (CPC‐M), Member of the German Center for Lung Research (DZL) Munich Germany; ^11^ Chair of Epidemiology, Institute for Medical Information Processing, Biometry and Epidemiology, Medical Faculty, Ludwig‐Maximilians‐Universität München Munich Germany; ^12^ Department of Internal Medicine B University Medicine Greifswald Greifswald Germany; ^13^ Institute for Community Medicine, University Medicine Greifswald Greifswald Germany; ^14^ Department of Medicine, Pulmonary and Critical Care Medicine University Medical Centre Marburg, Philipps‐University Marburg Marburg Germany; ^15^ German Centre for Lung Research (DZL) Marburg Germany; ^16^ Institute for Lung Health Giessen Germany; ^17^ German Centre for Infectious Disease Research (DZIF) Germany

**Keywords:** airflow limitation, COPD, KORA, lung function, SHIP, small airway dysfunction

## Abstract

**Background and Objective:**

Incident airflow limitation is frequently diagnosed at advanced stages. Identifying individuals at risk through primary care spirometry may enable earlier intervention, yet validated, pragmatic frameworks remain lacking.

**Methods:**

We applied a framework of three mutually exclusive spirometric at‐risk phenotypes, termed Three‐Phenotype Spirometry‐Based Identification (hereafter TriSpi), to 6123 participants from two population‐based cohorts: KORA (*n* = 1973, 3‐year Follow‐up, derivation) and SHIP (*n* = 4150, 5‐year Follow‐up, validation). TriSpi^+^ individuals were defined as meeting criteria for one of the three phenotypes: Early airflow limitation (EAL, FEV_1_/FVC > 0.7 and < 10th percentile or < 0.7 and > 5th percentile), small airway dysfunction (SAD) defined using FEF_50_‐ or FEF_75_‐based thresholds; and preserved‐ratio impaired‐spirometry (PRISm). Associations with incident airflow limitation were tested using Firth's regression.

**Results:**

EAL, PRISm, and SAD (defined using either FEF_50_ or FEF_75_‐based thresholds) were significantly associated with incident airflow limitation across cohorts and follow‐ups. TriSpi^+^ individuals accounted for 26%–36% of the population, identifying 74%–93% of future cases, while TriSpi^+^ was associated with a 10–25‐fold increase in risk. Negative predictive values exceeded 95% across definitions, and the number needed to screen among TriSpi^+^ individuals ranged from 7 to 13. EAL showed the strongest individual association (OR up to 53.2), while SAD was more common in younger adults.

**Conclusion:**

Combining definitions for EAL, PRISm, and SAD enables robust prediction of incident airflow limitation. TriSpi may serve as a scalable, pragmatic approach for early risk stratification in primary care, in absence of post‐BD spirometry.

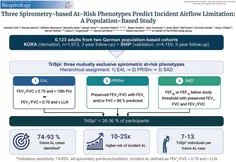

## Graphical abstract

Analysing the population‐based KORA and SHIP studies, three spirometric at‐risk subtypes (early airflow limitation, PRISm, and small airway dysfunction) were consistently associated with incident airflow limitation. Their combination in a conceptual framework identified most future cases with high negative predictive value, suggesting potential for early risk stratification in primary care settings.

## 1 Introduction

Chronic obstructive pulmonary disease (COPD) is the fourth leading global cause of death [1], yet is often diagnosed at late stages, limiting opportunities for early intervention. Consequently, increasing attention has been directed toward the identification of at‐risk phenotypes associated with airflow limitation (AL). In this context, the concept of *pre‐COPD* has been proposed to describe individuals without established AL who exhibit chronic respiratory symptoms or functional abnormalities associated with increased risk for future COPD [2]. Importantly, pre‐COPD denotes an at‐risk state rather than established disease.

However, translation into clinical practice remains challenging, as identification of at‐risk individuals is hindered by definitional controversy [3, 4], heterogeneity of spirometric markers, and lack of validated risk stratification frameworks. Additionally, limited availability of post‐bronchodilator (post‐BD) spirometry in primary care settings may further constrain pragmatic implementation, despite evidence that pre‐ and post‐bronchodilator (BD) spirometry shows similar prognostic value for long‐term mortality in subjects at risk for and with COPD [5].

Several at‐risk phenotypes have been proposed, including preserved‐ratio impaired spirometry (PRISm), early airflow limitation (EAL), small airway dysfunction (SAD) and chronic bronchitis. PRISm affects between 7% and 11% of adults [6, 7, 8], and has been linked to all‐cause mortality [7, 8]. EAL or discordant AL, defined by FEV_1_/FVC below 0.7 but above the lower limit of normal (LLN, defined as 5th percentile) may reflect an early or transitional phenotype [9]. Individuals with EAL have been shown to exhibit significantly lower mortality compared to patients whose FEV_1_/FVC is both below 0.7 and the LLN (concordant AL), yet exhibit accelerated lung function decline and are at high risk of progressing to concordant AL [10, 11]. Similar findings using alternative thresholds have been reported, with Tan et al. showing that an FEV_1_/FVC below the 10th percentile, termed subthreshold AL, improved discrimination for incident COPD [12], suggesting that the above mentioned discordant AL may not fully capture early disease risk. Small airway dysfunction (SAD), potentially indicating early stages of airway remodelling, is captured using mid‐exploratory flow metrics, for example, FEF_50_ or FEF_75_ [13, 14]. Kwon et al. demonstrated that z‐adjusted FEF_25‐75_ < −0.8435 (~16th percentile) was associated with incident AL, suggesting a quantifiable FEF‐based threshold for SAD [13]. By contrast, chronic bronchitis, defined by three‐month cough and sputum production but normal spirometry, represents a conceptually different, symptom‐defined entity, with variable prognostic value [9, 15]. Despite growing evidence linking spirometry‐based abnormalities to incident AL, and the known limitations of symptom‐based case finding in its prediction [16, 17], spirometry is currently only recommended in symptomatic individuals [18]. This practice may result in missed opportunities for earlier detection and intervention.

Here, we hypothesised that SAD and an expanded definition of EAL, including both individuals with subthreshold and discordant AL, identify individuals at risk for incident AL. We developed a spirometry‐based framework, called TriSpi (*Three‐Phenotype Spirometry‐Based Identification*), combining SAD, PRISm, and EAL, and evaluated its utility, sensitivity, and predictive value in two large population‐based cohorts.

## 2 Methods

### 2.1 Participants and Collected Data

We curated data from 6123 participants in two epidemiological research platforms, the ‘Cooperative Health Research in the Region of Augsburg’ (KORA) [19, 20] and the ‘Study of Health in Pomerania’ (SHIP) [21]. KORA was used for exploration, SHIP for independent validation. Both studies recruited randomly selected adults from the general population in southern (KORA) and northeastern (SHIP) Germany, who underwent interviews, physical examinations, and spirometry during baseline and follow‐up visits. From KORA, we included: KORA‐AGE (age 65–90 years, 3‐year follow‐up) and KORA‐F4 (age 41–62 years, 3‐ and 6‐year follow‐up). From SHIP, we used the SHIP‐START‐1 (age 25–86, 5‐, 10‐, and 15‐year follow‐up) and SHIP‐TREND‐0 cohort (age 20–81, 5‐year follow‐up).

Post‐bronchodilator (post‐BD) spirometry data were unavailable. Patients with baseline COPD, asthma or chronic lung conditions other than chronic bronchitis were excluded. AL was defined as FEV_1_/FVC < 0.7 and < LLN.

Four at‐risk phenotypes were defined for our analysis. PRISm was defined as FEV_1_ and/or FVC < 80% predicted in patients without AL. EAL was based on the definition provided by Çolak [9] but extended to incorporate two subgroups: (1) FEV_1_/FVC ≥ 0.7 but in the lower 10th percentile (z‐FEV_1_/FVC < −1.264), based on results by the tasmanian health study [12], and (2) patients with original EAL or discordant AL (FEV_1_/FVC < 0.7 and FEV_1_/FVC ≥ LLN). Multiple definitions for small airway dysfunction (SAD) were explored using FEF_50_ or FEF_75_, each defined as the respective FEF‐based marker below a threshold (e.g., FEF_75_‐SAD: z‐FEF_75_ < −0.2312) in patients without PRISm or EAL. FEF25‐75 thresholds from the KORA cohort were provided as supplementary analyses, but are not part of the final models, as they were unavailable in SHIP. Chronic bronchitis was defined as a history of expectoration and cough exceeding 3 months per year for at least 1 year and otherwise healthy respiratory status.

Healthy respiratory status was defined as the following: no chronic bronchitis history, FEV_1_/FVC > 0.7, z‐FEV_1_/FVC ≥ −1.2645 (10th percentile), FEV_1_ and FVC ≥ 80% predicted, and absence of SAD (e.g., for FEF_75_‐SAD: z‐FEF_75_ ≥ −0.2312).

### 2.2 Statistical and Machine Learning Analysis

Demographic and clinical characteristics were summarised per cohort and phenotype. Comparisons were performed using Wilcoxon's rank‐sum test, Fisher's exact test, or chi2 test (*gtsummary* v. 2) [22]. To compare the abundance of at‐risk phenotypes at baseline, we used chi2 tests, with Fisher's exact test as a post hoc test. Clinical utility of different TriSpi frameworks was assessed via sensitivity, specificity, positive and negative predictive values (PPV, NPV), the absolute and relative risk reduction (ARR, RRR), and the number needed to screen (NNS) in the general and the TriSpi^+^ population.

To assess the most important predictors for incident AL in the pooled KORA and SHIP cohorts, we trained a Random Forest classifier (*scikit‐learn*, v.1.6.1) to predict incident AL, with subsequent feature importance analysis. Validation was performed on a stratified 75/25% train‐test split. Addressing the class imbalance during training, the training dataset was imputed to handle missing data as a prerequisite for upsampling the minority class (no incident AL) using the Synthetic Minority Over‐sampling Technique (SMOTE [23]). This ensured a balanced class distribution during the training phase. Hyperparameters were optimised by random search 10‐fold cross‐validation, using Matthews Correlation Coefficient (MCC) as the scoring metric. Performance was evaluated on the validation set, including missing values, using accuracy, balanced F1 score, MCC, and area under the curve (AUC). Feature Importance Analysis was used to rank predictors of incident AL.

Associations with incident AL were assessed by Firth's penalised logistic regression (via *logistf*, v1.26) reducing bias due to low event counts, adjusting for age and smoking status, and excluding patients classifying as ‘Other’ (Table S1) to reduce covariate numbers. Additional adjustments for sex and smoking pack years (unavailable in SHIP‐Start‐1) were performed when event counts were sufficient (event/covariate ratio ≥ 5).

Receiver operating characteristic (ROC) analysis (*pROC*, v1.18) [24] and random effect meta‐analyses (*metafor*, v. 4.8) [25] assessed prognostic strength of FEF and at‐risk phenotypes. Predicted values and z‐scores were calculated using *PySpiro* (v.0.3.7) [26], applying GLI [27], and Luftibus reference equations (for FEF_25_ and FEF_50_) [28]. Data analysis was conducted in python (v.3.10) and R (v.4.2).

## 3 Results

We analysed 6123 participants across two studies, with two cohorts each: KORA (KORA‐F4: *n* = 1132, KORA‐AGE: *n* = 841) for exploration and SHIP (SHIP‐START: *n* = 1722, SHIP‐TREND: *n* = 2428, Table S1) for validation. KORA participants were older than those in SHIP (median 59 vs. 53, *p* < 0.001), while active smoking was more common in SHIP (22 vs. 14%). 41 (2.8%) cases of incident AL were observed in KORA and 92 (3.7%) in SHIP (Table 1). Feature importances from a random forest model (AUC: 0.87, MCC: 0.27) suggested FEV_1_/FVC as the strongest predictor of incident AL in the pooled KORA and SHIP cohorts, followed by small airway markers such as FEF_75_ and FEF_50_ (Figures S2–S4).

**TABLE 1:** Characteristics of the pooled KORA and SHIP datasets.

Characteristic	KORA, *N* = 1973^a^	SHIP, *N* = 4150^a^	*p*‐value^b^
Age [a]	59 (51, 73)	53 (41, 63)	< 0.001
BMI [kg/m^2^]	27.3 (24.6, 30.5)	27.5 (24.6, 30.7)	0.4
Sex			0.3
Male	955 (48%)	2064 (50%)	
Female	1018 (52%)	2086 (50%)	
Smoking Status			< 0.001
Current Smoker	280 (14%)	932 (22%)	
Former Smoker	744 (38%)	1521 (37%)	
Never Smoker	949 (48%)	1694 (41%)	
Smoking Pack Years	0 (0, 16)	0 (0, 9)	< 0.001
z‐FEV_1_ [GLI 2012]	0.43 (−0.26, 1.13)	0.18 (−0.51, 0.81)	< 0.001
z‐FVC [GLI 2012]	0.44 (−0.18, 1.08)	−0.08 (−0.70, 0.53)	< 0.001
z‐FEV_1_/FVC [GLI 2012]	−0.10 (−0.58, 0.38)	0.35 (−0.24, 1.00)	< 0.001
z‐FEF75 [GLI 2012]	0.49 (−0.01, 0.98)	0.56 (−0.04, 1.11)	0.015
z‐FEF25‐75 [GLI 2012]	0.07 (−0.50, 0.66)	—	
FEF50% predicted [Kuster 2008]	111 (89, 143)	113 (91, 139)	0.6
Incident AL at 3‐/5‐years Follow‐up	41 (2.8%)	92 (3.7%)	0.11
Incident AL at 6‐/10‐years Follow‐up	44 (5.1%)	65 (7.9%)	0.020

*Note:* Baseline and follow‐up data for all 6123 patients included in the study were given. Datasets represent pooled data from four German population‐based cohorts: KORA‐AGE (*n* = 841), KORA‐F4 (*n* = 1132), SHIP‐START (*n* = 1722, with SHIP‐START‐1 as baseline, as no spirometry was performed in SHIP‐START‐0), and SHIP‐TREND (*n* = 2428). All spirometry values were sampled pre‐bronchodilator. FEV_1_, FVC, FEV_1_/FVC, FEF_75_ and FEF_25‐75_ were adjusted using the GLI2012 reference equations [27]. FEF_50_ was adjusted to percent predicted using the LuftiBus reference equations [28]. Patients with baseline asthma, COPD or chronic lung conditions other than chronic bronchitis were excluded. Follow‐up data were available at 3 years for KORA‐AGE, 5 years for SHIP‐TREND, and 3 and 6 years for KORA‐F4. FU: Follow‐up. FEF_25‐75_ data were not available for SHIP at baseline. Smoking pack years were not available for baseline SHIP‐START. A representation of individual cohort data is given in Table S1.

^a^ Median (Q1, Q3); *n* (%).

^b^ Wilcoxon rank sum test; Pearson's Chi‐squared test.

### 3.1 Discriminative Ability of Small Airway Markers for Incident Airway Limitation

We applied ROC analysis to find thresholds for FEF_25_, FEF_50_, or FEF_75_ associated with 3‐year incident AL in the derivation (pooled KORA) cohort (Figure 1). FEF_50_ and FEF_25_ (percent predicted each) exhibited the highest AUC (80.5% and 80.4%) of the analysed small airway markers.

**FIGURE 1:**
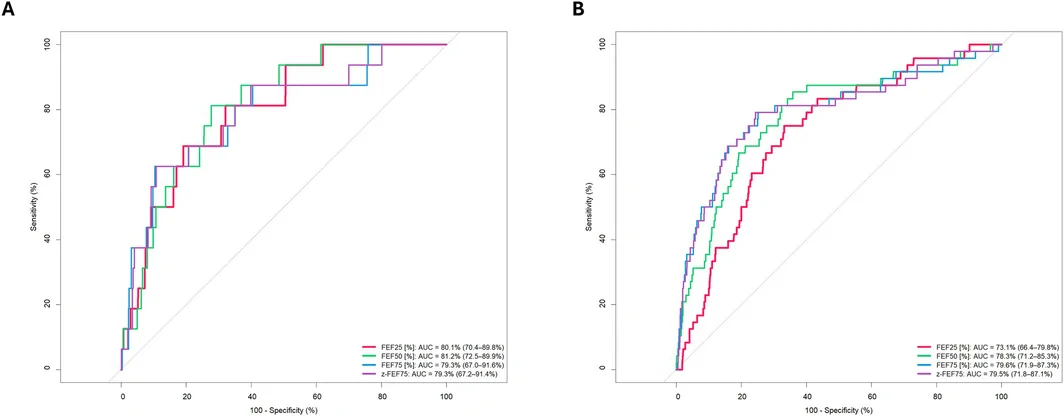
Discriminative ability of forced expiratory flow measures for incident AL in KORA and SHIP. Receiver operating characteristic (ROC) curves were generated for small airway markers, such as FEF_25_, FEF_50_, FEF_75_; in spirometrically healthy individuals (FEV/FVC > 10th percentile and > 0.7, FEV_1_ and FVC > 80% predicted and no history of chronic bronchitis). All values were adjusted for covariates using the GLI 2012 reference equations for FEF_75_, and the LuftiBus [28] reference equations for FEF_25_ and FEF_50_. AUC values were indicated alongside respective confidence intervals. (A) ROC curves for the pooled KORA cohort (3‐year incident AL). (B) ROC curves for the pooled SHIP cohort (5‐year incident AL). AL, airflow limitation.

We calculated Youden‐index‐optimised thresholds for FEF markers, based on our ROC analysis on the derivation cohort, comparing their prognostic strength for 3‐year incident AL (Table 2). The highest overall sensitivity (88.0%) was observed for a FEF_50_‐based threshold (% predicted, Luftibus reference equations [28]), while the highest specificity (89.4%) was seen for the FEF_75_‐based threshold (% predicted, adjusted using the GLI reference equations [27]).

**TABLE 2:** Small airway markers predict medium‐term incident AL in healthy individuals.

		Threshold	Sensitivity [%]	Specificity [%]	PPV	NPV	Accuracy
Pooled KORA (*n* = 1293^a^, 3‐year FU)	FEF_25_ [% pred.]	76.415	66.7	83.5	4.8	99.5	83.3
	FEF_50_ [% pred.]	96.67	88.0	72.6	3.5	99.7	72.7
	FEF_75_ [% pred.]	88.695	60.0	89.8	6.9	99.4	89.4
	z‐FEF_75_	−0.2312399	60.0	89.4	6.6	99.4	89.0
	FEF_25‐75_ [% pred.]	89.51	93.3	75.4	4.5	99.9	75.7
	z‐FEF_25‐75_	−0.3260781	93.3	75.9	4.6	99.9	76.1
	Kwon's z‐FEF_25‐75_ cutoff	−0.8435	47.1	93.3	8.5	99.2	92.7
Pooled SHIP (*n* = 2184^a^, 5‐year FU)	FEF_25_ [% pred.]	84.95	75.0	66.8	4.8	99.2	67.0
	FEF_50_ [% pred.]	107.715	85.4	64.2	5.1	99.5	64.7
	FEF_75_ [% pred.]	108.275	79.2	74.9	6.6	99.4	75.0
	z‐FEF_75_	0.1529761	79.2	75.6	6.8	99.4	75.7

*Note:* We evaluated the predictive utility of small airway markers (FEF_25_, FEF_50_, FEF_75_, and FEF_25‐75_), using ROC analysis [29] in healthy individuals at baseline, defined as absence of COPD and other at‐risk phenotypes (EAL, Chronic Bronchitis, PRISm). All values were adjusted using the GLI 2012 reference equations (FEF_25‐75_ and FEF_75_) or the LuftiBus [28] reference equations (FEF_25_ and FEF_50_). Pooled datasets from KORA and SHIP, as well as the whole dataset (KORA + SHIP) were evaluated. Thresholds were optimised using Youden's method, and the associated sensitivity, specificity, positive predictive value (PPV), negative predictive value (NPV), and accuracy reported. We further compared the thresholds determined by us to the −0.8435 cutoff for z‐FEF_25‐75_ proposed by Kwon and colleagues [13].

Abbreviations: AL, airflow limitation; FU, follow‐up.

^a^ Patients with healthy status at baseline only (no AL, PRISm, EAL or chronic bronchitis at baseline).

Consecutively, we explored two definitions of small airway dysfunction, based solely on either FEF_75_ or FEF_50_, for validation in SHIP: FEF_75_‐SAD (z‐FEF_75_ < −0.2312, ~the lower 40th percentile) and FEF_50_‐SAD (FEF_50_% predicted < 96.67).

### 3.2 At‐Risk Phenotypes in KORA and SHIP at Baseline

Prevalence of at‐risk phenotypes (EAL, PRISm, FEF_75_‐SAD/FEF_50_‐SAD and chronic bronchitis) differed by cohorts (Table S2). Overall, prevalence ranged between 26% and 34% in KORA (using either FEF_75_‐ or FEF_50_‐based definitions of SAD) and between 26% and 36% in SHIP. In KORA, the most frequent phenotype was FEF_50_‐SAD (21% of participants), followed by EAL (11%), FEF_75_‐SAD (7.9%) and PRISm (4.5%). Comparatively, higher frequencies of PRISm were observed in SHIP, while EAL was less prevalent (FEF_50_‐SAD: 22%, FEF_75_‐SAD: 12%, PRISm: 8.2%, EAL: 4.1%).

Prevalence of at‐risk phenotypes was stratified by age (Figure 2), with comparison of patients younger and older than 50 showing FEF_75_‐SAD to be significantly more frequent in younger individuals for both datasets (KORA: 15 vs. 5.4%, *p* < 0.0001, SHIP: 18 vs. 7.1%, *p* < 0.0001, Tables S3 and S4). Comparatively, FEF_50_‐SAD was more common in younger patients in KORA, but not in SHIP (KORA: 25% vs. 19%, *p* = 0.004; SHIP: 23% vs. 21%, *p* = 0.11).

**FIGURE 2:**
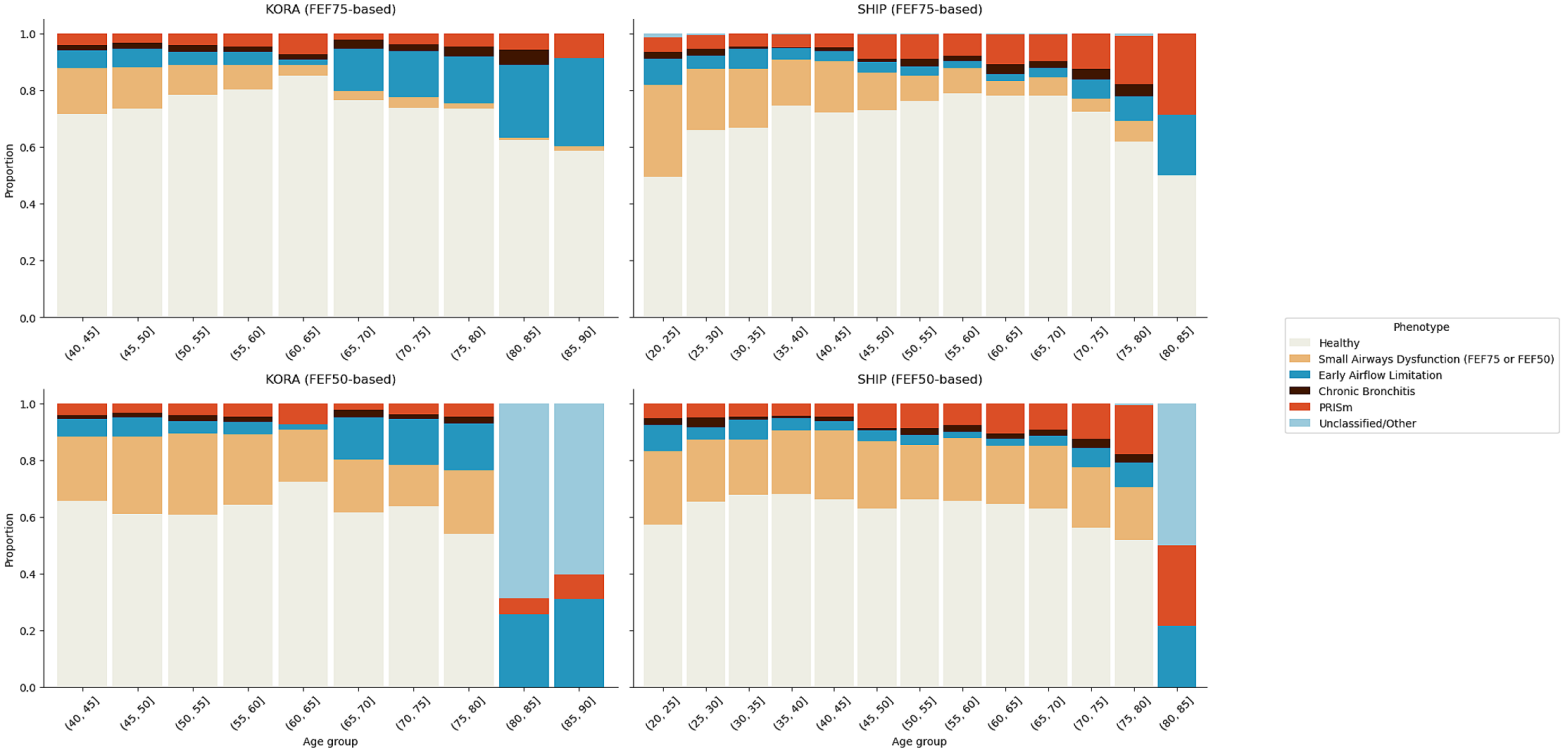
Age‐wise distribution of at‐risk phenotypes in the pooled KORA and SHIP cohorts. FEF_50_‐ and FEF_75_‐based thresholds for prediction of incident ALwere derived using ROC‐analysis of the pooled KORA cohort. Subsequently, two sets of at‐risk phenotype definitions were applied to the pooled KORA (derivation, *n* = 1973) and SHIP (validation, *n* = 4150) datasets, each containing EAL, PRISm, chronic bronchitis, and SAD (based either on FEF_75_ or FEF_50_). Here, we illustrate the age‐wise distribution of the at‐risk phenotypes in both datasets at baseline. Patients with available data for FEV_1_ and FVC but not for FEF metrics were classified as ‘Other’. Additionally, 144 individuals from the KORA dataset were older than 80 years, exceeding the valid range for the Luftibus equations, and were classified as ‘Other/Unclassified’ if not exhibiting an at‐risk phenotype. The gap in the KORA graph is explained by the relatively small overlap of the individual studies: KORA‐F4 (median age 52 years) and KORA‐AGE (median age 76 years). A table detailing the absolute and relative frequencies of at‐risk phenotypes is given in Table S2.

Similarly, EAL and PRISm differed by age, with EAL being more frequent in patients aged > 50 years in KORA (6.3% vs. 12%, *p* = 0.0002), but not in SHIP (4.5% vs. 3.8%, *p* = 0.2). PRISm was also more common in older participants in SHIP (5.7 vs. 10.0, *p* < 0.0001) but not in KORA (3.7% vs. 4.5%, *p* = 0.5), possibly due to lower baseline FEV_1_/FVC in the latter (Table 1).

Concordance between FEF markers was low in both cohorts, with less than half of SAD cases (KORA: 26%, SHIP: 30%) captured by more than one definition (Figure S5).

### 3.3 Association of At‐Risk Phenotypes With Incident AL

Subsequently we evaluated the association of individual at‐risk markers with incident ALin both cohorts. FEF_75_‐SAD was significantly associated with incident ALin the derivation (KORA, 3‐year FU, OR 9.21, 95% CI 2.93–30.07, *p* = 0.0002) and validation datasets (SHIP, 5‐year FU, OR 7.75, 95% CI 4.28–14.14, *p* < 0.0001, Table S5). Of the baseline FEF_75_‐SAD^+^ participants, 5.4 (KORA)–8.6 (SHIP) % exhibited AL at follow‐up. FEF_50_‐SAD showed similar associations (KORA: OR 9.68, 95% CI 2.86–40.73, *p* = 0.0002; SHIP: OR 9.40, 95% CI 5.0–18.37, *p* < 0.0001, Table S6), with 2.8 (KORA)–6.4 (SHIP) % developing AL.

For PRISm, significant associations were found in SHIP (5.7% developing COPD, OR 5.12, CI 2.24–10.86), but not in KORA (*p* = 0.1, Tables S5 and S6). EAL was strongly associated with incident AL, with 19%–37% (KORA, SHIP) of baseline positive participants exhibiting disease at follow‐up, and regression models showing very high OR in both cohorts (KORA: OR 53.2, 95% CI: 21.7–155.3, *p* < 0.0001; SHIP: OR 46.36, 95% CI 25.6–85.8, *p* < 0.0001). Chronic Bronchitis consistently failed to exhibit associations with incident ALand was therefore excluded from further analysis.

As our definition of EAL encompassed both patients with discordant airflow limitation (FEV_1_/FVC < 0.7 but > 5th percentile (LLN)) and those with FEV_1_/FVC > 0.7 but < 10th percentile, we conducted sensitivity analyses to test these groups separately, finding significant associations with incident AL for both groups across all cohorts (Table S7).

Continuing our sensitivity analyses, we tested the association between the investigated at‐risk phenotypes and incident AL for longer follow‐ups (KORA‐F4: 6‐year, SHIP‐START: 5‐, 10‐, 15‐year FU), showing that FEF_75_‐ and FEF_50_‐SAD, FEF_25‐75_‐SAD, PRISm and EAL were consistently associated with incident ALin all investigated follow‐ups (Tables S8–S10).

### 3.4 Clinical Utility of Definition Frameworks in Predicting Incident Airflow Limitation

Last, we assessed the clinical utility of the three spirometric at‐risk phenotypes (SAD, PRISm, and EAL) as a whole, integrating them into a composite framework, which we refer to as TriSpi. We systematically evaluated TriSpi in both derivation and validation cohorts, varying only the SAD definition using different FEF‐based thresholds.

AL incidence was low in both datasets (KORA, 3‐year FU, 2.8%, SHIP, 5‐year FU, 3.7%). Across all TriSpi variants, positive predictive values (PPVs) remained below 20%, whereas negative predictive values (NPVs) consistently exceeded 95% (Table 3). Despite the low incidence, TriSpi^+^ status identified 88%–93% of future COPD cases in KORA, and 74%–83% in SHIP. Compared to TriSpi^−^ participants, TriSpi^+^ participants showed a 21–25‐fold increased incident AL risk in KORA, and a 10–11‐fold increased risk in SHIP. The NNS to detect one future case ranged from 32 to 44 in the general population and from 7 to 13 in the TriSpi^+^ group. Notably, defining SAD as FEF_75_‐SAD and/or FEF_50_‐SAD achieved the highest diagnostic coverage, with a relative risk reduction (RRR) of 98% in KORA and 93% in SHIP.

**TABLE 3:** Clinical value of *TriSpi* variants for prediction of incident AL in the general population.

Cohort	*TriSpi* Version (EAL, PRISm, and …)	Incidence of AL: *n* (%)		PPV	NPV	RR	ARR (TriSpi^−^)	RRR (TriSpi^−^)	NNS (General Population)	NNS (TriSpi^+^)
		TriSpi^+^	TriSpi^−^							
Pooled‐KORA (3‐y FU)	FEF_25‐75_‐SAD	34 (12.54)	7 (0.58)	12.5	99.4	21.4	−11.87	−95.33	43.4	8.0
	FEF_75_‐SAD	36 (11.2)	5 (0.43)	11.2	99.6	25.8	‐ 10.75	−96.12	41.0	8.9
	FEF_50_‐SAD	38 (7.6)	3 (0.31)	7.4	99.7	23.6	−7.05	−95.76	38.8	13.6
	FEF_50_‐SAD *and* FEF_75_‐SAD	35 (12.82)	6 (0.5)	12.8	99.5	25.7	−12.32	−96.11	42.2	7.8
	FEF_50_‐SAD *or* FEF_75_‐SAD	39 (6.90)	2 (0.14)	6.9	99.8	49.3	−6.76	97.97	37.8	14.5
Pooled‐SHIP (5‐y FU)	FEF_75_‐SAD	68 (12.64)	24 (1.24)	12.6	98.8	10.2	−11.40	−90.20	36.4	7.9
	FEF_50_‐SAD	76 (10.01)	16 (0.93)	10.0	99.1	10.7	−9.08	−90.69	32.6	10.0
	FEF_50_‐SAD *and* FEF_75_‐SAD	67 (15.19)	24 (1.23)	15.2	98.8	12.4	−13.96	−91.91	36.9	6.6
	FEF_50_‐SAD *or* FEF_75_‐SAD	77 (9.0)	15 (0.63)	9.0	99.1	14.3	−8.36	92.99	32.11	11.1

*Note:* We applied different variants of the TriSpi framework to the pooled derivation (KORA) and validation (SHIP) cohorts. TriSpi is a set of three mutually exclusive definitions for spirometric at‐risk phenotypes, encompassing early airflow limitation (EAL), preserved‐ratio impaired‐spirometry (PRISm), and small airway dysfunction (SAD). We systematically varied the definition used for SAD, comparing their clinical utility, using positive and negative predictive values (PPV, NPV), relative risk (RR), absolute risk reduction (ARR) and relative risk reduction (RRR), as well as the number needed to screen (NNS) to identify one case of incident ALin the general population as well as the TriSpi^+^‐population. NNS was calculated as (Prevalence(TriSpi)_GeneralPopulation_ * Incidence(COPD)_TriSpi‐Patients_)^−1^ for the general population and (Incidence(COPD)_TriSpi‐Patients_)^−1^ for the TriSpi population.

Abbreviation: AL, airflow limitation.

We further validated the frameworks using extended follow‐up data (Table S11). In KORA‐F4 (6‐year follow‐up, 5.1% incidence), FEF_75_‐ and FEF_50_‐based TriSpi identified > 80% of future cases, with RRR of 12%–19% in TriSpi^−^. In SHIP‐START (10‐year FU: 8.0% incidence), FEF_50_ maintained good predictive performance (83% of future cases identified), while FEF_75_ identified only 43%. SHIP‐START's 15‐years FU (4.2%) was excluded due to excessive loss to follow‐up in the absence of mortality data. Across all timepoints, combined SAD definitions increased sensitivity when used inclusively (FEF_50_ or FEF_75_) and specificity when used jointly (FEF_50_ and FEF_75_).

Additionally, we tested combinations of two and three combinations, with highest sensitivity when combining three at‐risk phenotypes, both KORA and SHIP (Table S12).

Finally, we derived simplified, clinically applicable thresholds for SAD using ROC analysis in the derivation cohort, and validated them in SHIP. For each FEF marker, we defined (1) high sensitivity rule‐out threshold (> 90%), (2) an intermediate cutoff with > 80% specificity, and (3) a high‐specificity referral threshold with (comparatively) higher PPV. These cutoffs were harmonised across FEF_50_ and FEF_75_ to enhance usability in routine primary care (Table 4).

**TABLE 4:** Exploring a three‐step definition of small airway dysfunction for prediction of incident AL in the general population.

Cohort	SAD definition		Incidence of AL: *n* (%)		Sensitivity	Specificity	PPV	NPV	RR	ARR (TriSpi^−^)	RRR (TriSpi^−^)	NNS (General Population)	NNS (TriSpi^+^)
			TriSpi^+^	TriSpi^−^									
Pooled‐KORA (3‐y FU)	FEF_75_	< 110%	37 (6.60)	4 (0.44)	90.2	63.5	6.6	99.6	15.1	−6.16	−93.37	39.9	15.2
		< 80%	33 (12.74)	8 (0.66)	80.5	84.3	12.7	99.3	19.4	−12.08	−94.84	44.7	7.8
		< 50%	29 (15.10)	12 (0.93)	70.7	88.6	15.1	99.1	16.2	−14.17	−93.81	50.9	6.6
	FEF_50_	< 110%	39 (5.58)	2 (0.26)	95.1	54.0	5.6	99.7	21.7	−5.32	95.39	37.8	17.9
		< 80%	33 (10.48)	8 (0.69)	80.5	80.3	10.5	99.3	15.2	−9.79	−93.42	44.7	9.5
		< 50%	29 (15.03)	12 (0.94)	70.7	88.6	15.0	99.1	16.1	−14.09	−93.78	50.9	6.7
Pooled‐SHIP (5‐y FU)	FEF_75_	< 110%	80 (9.11)	12 (0.75)	87.0	66.5	9.1	99.2	12.1	−8.36	−91.75	30.9	11.0
		< 80%	64 (15.61)	28 (1.36)	69.6	85.5	15.6	98.6	11.5	−14.25	−91.31	38.7	6.4
		< 50%	47 (17.41)	45 (2.04)	51.6	90.6	17.4	98.0	8.5	−15.37	−88.28	52.7	5.7
	FEF_50_	< 110%	85 (7.56)	7 (0.52)	92.4	56.4	7.6	99.5	14.6	−7.04	−93.15	29.1	13.2
		< 80%	58 (13.09)	34 (1.67)	63.0	83.8	13.1	98.3	7.8	−11.42	−87.22	42.7	7.6
		< 50%	44 (17.25)	48 (2.16)	47.99	91.1	17.3	97.8	8.0	−15.09	−87.47	56.2	5.8
	FEF_50_ or FEF_50_	< 110%	87 (6.64)	5 (0.22)	94.6	48.7	6.6	99.6	30.4	−6.42	−96.71	28.4	15.1

*Note:* We aimed to radically simplify our cutoffs for definition of small airway dysfunction, using ROC analysis of the derivation (KORA) cohort to choose new thresholds for FEF_50_ and FEF_75_, which were then harmonised. We chose three thresholds for each marker: (1) high sensitivity rule‐out (> 90%), (2) intermediate with > 80% specificity, and (3) high‐specificity referral with (comparatively) higher PPV. Harmonising these cutoffs yielded relatively simpler sequences of 50%, 80%, and 110% predicted for both FEF‐markers. We explored the predictive performance of this three‐step approach by applying the respective TriSpi framework (using FEF < 110%, < 80% or < 50% as definitions for small airway dysfunction) across both cohorts. We compared clinical utility using positive and negative predictive values (PPV, NPV), relative risk (RR), absolute risk reduction (ARR) and relative risk reduction (RRR), as well as the number needed to screen (NNS) to identify one case of incident AL in the general population as well as the TriSpi^+^‐population. NNS was calculated as (Prevalence(TriSpi)_GeneralPopulation_ * Incidence(AL)_TriSpi‐Patients_)^−1^ for the general population and (Incidence(AL)_TriSpi‐Patients_)^−1^ for the TriSpi population.

Abbreviation: AL, airflow limitation.

In the validation cohort, FEF_50_ and FEF_75_ rule‐out thresholds achieved NPVs of 99.2%–99.5% (RRR: 93%–95%), and a combined rule‐out (FEF_50_ and FEF_75_) achieved 99.6% NPV (RRR 97%). Referral thresholds yielded PPVs up to 15% with an NNS of 6 among TriSpi^+^ individuals. While exploratory, these thresholds may serve as a basis for future risk stratification initiatives in primary care.

## 4 Discussion

In this multi‐cohort study, we developed and validated a spirometry‐based framework (TriSpi) encompassing three, non‐overlapping, spirometry‐based at‐risk phenotypes (SAD, EAL, PRISm), each of which consistently exhibited associations with incident AL across cohorts and follow‐ups. Despite the low incidence of AL (2.8%–3.7%), TriSpi^+^ identified the majority of future cases (74%–93%), showed a 10–25‐fold increase in risk compared to TriSpi^−^, and yielded NPVs > 95% across all definitions. The NNS ranged from 32 to 44 in the general population, and from 6 to 13 among TriSpi^+^ individuals, suggesting that spirometry‐first risk stratification may be feasible and effective in real‐world primary care.

In current routine practice, spirometry is only recommended for symptomatic individuals [18]. Yet, symptoms alone are insufficient for identifying undiagnosed COPD [16], as a substantial proportion of individuals with pre‐ or early COPD are asymptomatic or may minimise symptoms through reduced activity levels [17]. Here, chronic bronchitis was included as an exploratory, symptom‐based at‐risk phenotype, which, due to inconsistent associations, was later excluded from the final TriSpi framework, which is now fully spirometry‐based. By contrast, TriSpi offers a relatively easy approach not requiring symptom assessment, imaging, or imaging, thereby addressing a key gap to current case‐finding paradigms. Given the widespread availability of pre‐BD spirometry in primary care and occupational health, TriSpi may help shift detection of airflow limitation toward earlier identification of at‐risk individuals, particularly when post‐bronchodilator spirometry is not available.

Among the individual phenotypes, EAL emerged as the most powerful and consistent predictor of COPD risk, particularly in older adults. Our definition for EAL was based on work by Colak and colleagues (FEV_1_/FVC > 0.7 and < 5th percentile) [9]. However, based on results from the Tasmanian Health Study [12], we broadened the threshold to the 10th percentile and added a second group of individuals (discordant airflow limitation (FEV_1_/FVC < 0.7 and > LLN)). Using this two‐group approach, we aimed to harmonise fixed‐ratio (GOLD [30]) and LLN‐based (ATS/ERS [31, 32]) criteria, capturing transitional states of airflow limitation across the lifespan. Individuals with EAL had incidence rates of up to 37% at follow‐up, and both subgroups were associated with incident ALin supplementary analyses, supporting prior findings that discordant airflow limitation is associated with accelerated lung function decline and eventual disease conversion [10, 11, 33]. While this should not delay screening for at‐risk states now, future research is needed to delineate whether EAL, which possibly represents a transitional state with different prognosis than already established COPD, is best described as pre‐ [2], latent [34], or early [35] COPD.

SAD, captured via FEF_75_ or FEF_50_ thresholds, was the most prevalent TriSpi component and more common in younger adults. There have been long‐standing concerns over mid‐expiratory flow markers which have been shown to exhibit considerable variability [36]. However, our findings reaffirm the predictive value of mid‐expiratory flow markers for prediction of incident AL when combined with preserved FEV_1_, FVC and FEV_1_/FVC, despite inter‐study heterogeneity. Importantly, we demonstrate that concordance between different FEF‐metrics increases COPD risk, in line with recent studies suggesting that marker overlap increases specificity [37].

PRISm showed weaker associations than EAL or SAD, likely reflecting its phenotypic heterogeneity, which may encompass both restrictive and early obstructive patterns [6]. However, we acknowledge that our broader spirometry criteria (aligned with Colak [9]) differ from those used in studies like Rotterdam, and that PRISm, aside from prediction of airflow limitation, is associated with increased mortality [7]. Further, PRISm remained significantly associated with COPD risk in the validation cohort and delineates an important at‐risk state warranting early identification and follow‐up.

A major strength of our study lies in its focus on clinical applicability. We derived and validated simplified thresholds for FEF_50_ and FEF_75_, stratified into (i) high‐sensitivity rule‐out cutoffs, (ii) intermediate thresholds for monitoring, and (iii) high‐specificity referral thresholds. These were harmonised to support practical implementation in primary care. In the validation cohort, rule‐out thresholds achieved NPVs of up to 99.6%, and relative risk reductions of 93%–97%, while referral thresholds yielded PPVs of up to 17%, and a NNS of as low as 6 among TriSpi^+^ individuals. Although PPVs rank low, partially due to the low incidence of COPD, a potential TriSpi^+^ status in primary care would indicate closer follow‐up, post‐BD spirometry, comorbidity optimisation or pneumologist referral, none of which constitutes a grave medical intervention requiring higher PPVs.

Several limitations apply to our study. First, we used pre‐bronchodilator spirometry, which may overestimate COPD incidence due to reversibility in Asthma. However, this reflects the reality of most primary care settings and enhances real‐world applicability. Second, we could not account for competing risks such as mortality, and data granularity did not allow application of dedicated time‐to‐event (e.g., Cox) models. Third, the number of incident AL cases was limited, as expected in a general population cohort with intermediate follow‐up, which may reduce precision and limit generalisability, despite the use of robust regression and machine learning approaches. In addition, symptom‐related data (e.g., wheeze; chronic airways assessment test, CAAT) were not consistently available across cohorts and were therefore not incorporated into the definition of healthy respiratory status. Smoking pack‐years data were also missing in a subset of the SHIP dataset (SHIP‐START). Fourth, we could not systematically assess the persistence of incident airflow limitation, as repeated follow‐up spirometry was not available in a harmonised manner across cohorts. Finally, we lacked imaging or biomarker data to further phenotype disease progression. Nonetheless, consistent associations across cohorts, time points and spirometry markers underscore the robustness of our findings.

TriSpi offers a simple, reproducible, and clinically applicable framework to identify individuals at risk in primary care, using standard spirometry. While spirometric abnormalities such as PRISm, EAL, and SAD have been previously associated with incident AL, our study is, to our knowledge, the first to integrate these into a unified, mutually exclusive framework and to validate this approach across two independent cohorts. By identifying high‐risk individuals with high sensitivity and low NNS, TriSpi may support targeted referral and resource allocation. While prospective validation and integration with imaging or molecular biomarkers are needed, our results suggest that, even on the scale of population‐level cohorts, early spirometry changes, particularly SAD or EAL, can serve as actionable warning before AL becomes clinically apparent.

## Author Contributions

**Margit Heier:** resources, project administration, writing – review and editing. **Max Kutzinski:** writing – original draft, writing – review and editing, methodology, investigation. **Gernot Rohde:** writing – review and editing, project administration, resources, supervision. **Wilhelm Bertrams:** conceptualization, project administration, resources, writing – original draft, writing – review and editing. **Ralf Ewert:** project administration, resources, writing – review and editing. **Stefan Karrasch:** writing – review and editing, writing – original draft, conceptualization, investigation, methodology, validation, visualization, project administration, resources, supervision. **Mustafa Abdo:** writing – review and editing, writing – original draft. **Dominik Heider:** project administration, resources, supervision, software, writing – review and editing. **Claus F. Vogelmeier:** project administration, resources, supervision, writing – review and editing. **Hendrik Pott:** conceptualization, investigation, writing – original draft, writing – review and editing, methodology, validation, visualization, software, formal analysis, project administration, data curation, resources. **Annette Peters:** supervision, resources, project administration, writing – review and editing. **Beate Stubbe:** project administration, resources, writing – review and editing. **Henry Völzke:** project administration, resources, writing – review and editing. **Anne Obst:** resources, project administration, writing – review and editing. **Bernd Schmeck:** conceptualization, investigation, funding acquisition, writing – review and editing, visualization, validation, methodology, project administration, resources, supervision. **Roman Martin:** conceptualization, investigation, methodology, resources, formal analysis, software, validation, visualization, writing – review and editing, writing – original draft.

## Funding

The KORA study was initiated and financed by the Helmholtz Zentrum München—German Research Center for Environmental Health, which is funded by the German Federal Ministry of Education and Research (BMBF) and by the State of Bavaria. Data collection in the KORA study is done in cooperation with the University Hospital of Augsburg. The KORA‐Age project was financed by the German Federal Ministry of Education and Research (BMBF FKZ 01ET0713 and 01ET1003A) as part of the ‘Health in old age’ program.

This study was funded by the German Federal Ministry of Research, Technology and Space (BMFTR) as part of the PerMed‐COPD Consortium (grant number 01EK2203A). This work was further supported by the German Center for Lung Research (DZL), grant number 82DZL073C2.

## Ethics Statement

All participants provided written informed consent, and the study methods were approved by the responsible ethics committee of the Bavarian Chamber of Physicians (EK No 06068 (F4 + FF4), EK No 10024(F4L), EK No 06068 (FF4), EK No 08064 (Age1 + Age2)).

## Conflicts of Interest

H.P. reports travel grants and support for meeting attendance by AstraZeneca. H.P. reports board membership in the scientific advisory board of the German Lung Foundation. R.E. reports consulting fees by Boerhinger Ingelheim, AOP and Lung Pacer, payment for lectures by AstraZeneca, Janssen and BerlinChemie, support for attending meetings and/or travel by Novartis and OMT, board membership at LungPacer, and a fiduciary role in AOP. D.H. reports payment for lectures by Sanofi. G.R. reports personal fees from Astra Zeneca, Atriva, Boehringer Ingelheim, GSK, Insmed, MSD, Sanofi, Novartis and Pfizer for consultancy during advisory board meetings. G.R. reports personal fees from Astra Zeneca, Berlin Chemie, BMS, Boehringer Ingelheim, Chiesi, Essex Pharma, Grifols, GSK, Insmed, MSD, Roche, Sanofi, Solvay, Takeda, Novartis, Pfizer and Vertex for lectures. G.R. reports a position as chairman of the CAPNETZ executive board. C.V: reports grants or contracts from the German ministry of education and science (BMBF), AstraZeneca, Boehringer Ingelheim, Chiesi, CLS Behring, GlaxoSmithKline, Grifols and Novartis. C.V. reports consulting fees by Aerogen, AstraZeneca, Boehringer Ingelheim, CSL Behring, Chiesi, GlaxoSmithKline, Insmed, Menarini, Novartis, Nuvaira, Roche and Sanofi, as well as payments or honoraria for lectures by Aerogen, AstraZeneca, Boehringer Ingelheim, CSL Behring, Chiesi, GlaxoSmithKline, Insmed, Menarini, Novartis, Roche and Sanofi. B.S. reports grants or contracts from CSL Behring and the German ministry of Health (BMG). R.M., W. B., M.A., M.H., A. P., B.S., M. K., A. O., H.V. and S.K. report nothing to disclose.

## Supporting information


**Table S1:** Characteristics of the pooled KORA and SHIP datasets. Baseline and follow‐up data for all 6123 patients included in the study were given. Datasets were generated from four German population‐based cohorts: KORA‐AGE (*n* = 841), KORA‐F4 (*n* = 1132), SHIP‐START (*n* = 1722, with SHIP‐START‐1 as baseline, as no spirometry was performed in SHIP‐START‐0), and SHIP‐TREND (*n* = 2428). All given spirometry values were sampled pre‐bronchodilator. FEV_1_, FVC, FEV_1_/FVC, FEF75 and FEF25‐75 were adjusted using the GLI2012 reference equations. FEF50 was adjusted to percent predicted using the LuftiBus reference equations^31^. Patients with baseline Asthma, COPD or chronic lung conditions other than chronic bronchitis were excluded. Follow‐up data were available at 3 years for KORA‐AGE, 5 years for SHIP‐TREND, and 3 and 6 years for KORA‐F4. FU: Follow‐up. FEF_25‐75_ data were not available for SHIP‐START or SHIP‐TREND at baseline. Smoking pack years were not available for baseline SHIP‐START. AL: Airflow limitation.
**Table S2:** Prevalences of at‐risk phenotypes in the pooled exploration (KORA) and validation (SHIP) datasets. Three frameworks of at‐risk phenotypes (early airflow limitation, EAL, preserved‐ratio impaired‐spirometry, PRISm) were applied to KORA and SHIP, each varying by the used small airway dysfunction (SAD) phenotype. In total, three definitions for small airway dysfunction (SAD) were explored: FEF_25‐75_‐SAD, FEF_50_‐SAD and FEF_75_‐SAD. Specifically, these definitions classified individuals as having SAD if FEF_25‐75_, FEF_50_, or FEF_75_ were below the Youden‐optimised threshold, derived in the KORA dataset (in absence of EAL or PRISm: FEV_1_/FVC > 0.7 and > 10th percentile, FEV_1_ and FVC > 80% predicted). For FEF_25‐75_ and FEF_75_, thresholds were applied to the respective^30^, while the FEF_50_‐threshold was applied to the percent predicted ratio (no z‐scores available) from the LuftiBus equations^31^. FEF_25‐75_ were unavailable for baseline SHIP and are only reported for KORA. Patients with available data for FEV_1_ and FVC but not for FEF metrics were classified as ‘Other’. Additionally, 144 individuals from the KORA dataset were older than 80 years, exceeding the valid range for the Luftibus equations, and were classified as ‘Other’.
**Table S3:** Prevalences of at‐risk phenotypes in patients younger and older than 50 years in KORA. Baseline prevalence of at‐risk phenotypes was compared between patients aged up to 50 years, and patients older than 50, using Pearson's chi2 test. Post hoc tests to compare individual subgroups were performed using Fisher's exact test.
**Table S4:** Prevalences of at‐risk phenotypes in patients younger and older than 50 years in SHIP. Baseline prevalence of at‐risk phenotypes was compared between patients aged up to 50 years, and patients older than 50, using Pearson's chi2 test. Post hoc tests to compare individual subgroups were performed using Fisher's exact test.
**Table S5:** Association of TriSpi‐FEF_75_ phenotypes with 3−/5‐year incident AL in the pooled KORA and SHIP cohorts. The Table reports cases with and without incident ALover 3‐ and 5‐ year follow‐up, stratified by baseline at‐risk phenotypes in the pooled KORA and SHIP cohorts. Due to the low event count, we used Firth's penalised logistic regression and limited the number of covariates to age, smoking status, sex, and the displayed at‐risk phenotypes, to receive an acceptable Event/Covariate ratio. Covariates, Covariate/Event ratios, Odds ratios (OR), 95% confidence intervals (CI) and *p*‐values are reported for all models. Regression Models were further adjusted for smoking pack years, which was not available for baseline SHIP‐START, and thus not included in the pooled SHIP model. Patients classified as ‘Other’ and were excluded from the regression model to further shrink the number of covariates. AL: Airflow limitation.
**Table S6:** Association of TriSpi‐FEF_50_ phenotypes with 3‐/5‐year incident ALin the pooled KORA and SHIP cohorts. The Table reports cases with and without incident AL over 3‐ and 5‐ year follow‐up, stratified by baseline at‐risk phenotypes in the pooled KORA and SHIP cohorts. Due to the low event count, we used Firth's penalised logistic regression and limited the number of covariates to age, smoking status, sex, and the displayed at‐risk phenotypes, to receive an acceptable Event/Covariate ratio. Covariates, Covariate/Event ratios, Odds ratios (OR), 95% confidence intervals (CI) and *p*‐values are reported for all models. Regression Models were further adjusted for smoking pack years, which was not available for baseline SHIP‐START, and thus not included in the pooled SHIP model. Patients classified as ‘Other’ and were excluded from the regression model to further shrink the number of covariates. AL: Airflow limitation.
**Table S7:** Both Discordant Airflow Limitation and Early Airflow Limitation with exceeded fixed‐ratio criterion predict incident AL. As part of our sensitivity analyses, we split the EAL group into patients with discordant airflow limitation (FEV_1_/FVC < 0.7 but > 5th percentile (LLN)) and those with FEV_1_/FVC > 0.7 but < 10th percentile. Associations with incident AL over 3‐ and 5‐ year follow‐up were investigated using Firth's penalised logistic regression. Covariates, Covariate/Event ratios, Odds ratios (OR), 95% confidence intervals (CI) and *p*‐values are reported for all models. Regression Models were further adjusted for smoking pack years, which was not available for baseline SHIP‐START, and thus not included in the pooled SHIP model. Patients classified as ‘Other’ and were excluded from the regression model to further shrink the number of covariates. AL: Airflow limitation.
**Table S8:** Association of TriSpi‐FEF_75_ phenotypes with 6‐/10‐/15‐year incident AL in the pooled KORA and SHIP cohorts. The Table reports cases with and without incident AL over 6‐, 10‐ and 15‐year follow‐up, stratified by baseline at‐risk phenotypes in the pooled KORA and SHIP cohorts. Due to the low event count, we used Firth's penalised logistic regression and limited the number of covariates to age, smoking status, sex, and the displayed at‐risk phenotypes, to receive an acceptable Event/Covariate ratio. Covariates, Covariate/Event ratios, Odds ratios (OR), 95% confidence intervals (CI) and *p*‐values are reported for all models. Regression Models were further adjusted for smoking pack years, which was not available for baseline SHIP‐START, and thus not included in the pooled SHIP model. Patients classified as ‘Other’ and were excluded from the regression model to further shrink the number of covariates. AL: Airflow limitation.
**Table S9:** Association of TriSpi‐FEF_50_ phenotypes with 6‐/10‐/15‐year incident AL in the pooled KORA and SHIP cohorts. The Table reports cases with and without incident AL over 6‐, 10‐ and 15‐year follow‐up, stratified by baseline at‐risk phenotypes in the pooled KORA and SHIP cohorts. Due to the low event count, we used Firth's penalised logistic regression and limited the number of covariates to age, smoking status, sex, and the displayed at‐risk phenotypes, to receive an acceptable Event/Covariate ratio. Covariates, Covariate/Event ratios, Odds ratios (OR), 95% confidence intervals (CI) and *p*‐values are reported for all models. Regression Models were further adjusted for smoking pack years, which was not available for baseline SHIP‐START, and thus not included in the pooled SHIP model. Patients classified as ‘Other’ and were excluded from the regression model to further shrink the number of covariates. AL: Airflow limitation.
**Table S10:** Association of TriSpi‐FEF_25‐75_ phenotypes with 3‐/6‐year incident AL in KORA. The Table reports cases with and without incident AL over 3‐ and 6‐ year follow‐up, stratified by baseline at‐risk phenotypes in the KORA cohort. Two models were generated, one for the pooled KORA cohort (3‐year FU), and one for the KORA‐F4 cohort, which had a second follow up (6‐year FU). We used Firth's penalised logistic regression and limited the number of covariates to age, smoking status, sex, and the displayed at‐risk phenotypes, to receive an acceptable Event/Covariate ratio. Covariates, Covariate/Event ratios, Odds ratios (OR), 95% confidence intervals (CI) and *p*‐values are reported for all models. Patients classified as ‘Other’ and were excluded from the regression model to further shrink the number of covariates. AL: Airflow limitation.
**Table S11:** Evaluating Variants of *TriSpi* for prediction of incident AL at longer follow‐ups. We applied different variants of the TriSpi framework to the subsets of the KORA and SHIP cohorts with longer follow‐up to evaluate their utility for prediction of incident ALat longer follow‐ups. TriSpi is a set of three mutually exclusive definitions for spirometric at‐risk phenotypes, encompassing early airflow limitation (EAL), preserved‐ratio impaired‐spirometry (PRISm) and small airway dysfunction (SAD). We systematically varied the definition used for SAD, comparing their clinical utility, using positive and negative predictive values (PPV, NPV), relative risk (RR), absolute risk reduction (ARR) and relative risk reduction (RRR), as well as the number needed to screen (NNS) to identify one case of incident ALin the general population as well as the TriSpi^+^‐population. NNS was calculated as (Prevalence(TriSpi)_GeneralPopulation_ * Incidence(AL)_TriSpi‐Patients_)^−1^ for the general population and (Incidence(AL)_TriSpi‐Patients_)^−1^ for the TriSpi population. AL: Airflow limitation.
**Table S12:** Benchmarking combinations of two at‐risk phenotypes against the all three at‐risk phenotypes. We assessed whether using three at‐risk phenotypes (early airflow limitation, preserved‐ratio impaired‐spirometry, and small airway dysfunction), provided benefit over using two at‐risk phenotypes. Combinations of two at‐risk phenotypes were applied to both the KORA (3‐year follow‐up, 1476 patients) and SHIP (5‐year follow‐up, 2475 patients) cohorts. Sensitivity, specificity, positive predictive value (ppv) and negative predictive value (npv), were calculated for each combination. Early airflow limitation: EAL, preserved‐ratio impaired‐spirometry: PRISm, small airway dysfunction based on FEF50: FEF50‐SAD, small airway dysfunction based on FEF75: FEF75‐SAD.
**Figure S1:** Included Cohorts, patients and respective follow‐up times. The given figure represents the patients in the original datasets of the four cohorts (KORA‐AGE, KORA‐F4, SHIP‐TREND and SHIP‐1), the numbers in the curated datasets, and the number of participants in the respective follow‐up visits. Patients with Asthma, COPD or chronic lung disease at baseline were excluded, their numbers indicated by the respective cohort. Loss to Follow‐up was similarly represented (*pt*.: Patients). No mortality data for dissection of Loss to Follow‐up were available for this analysis. In KORA‐AGE and ‐F4 patients completed a 3‐years follow‐up, plus an additional 6‐year follow‐up in KORA‐F4. Follow‐ups in both SHIP studies generally adhered to a 5‐year interval. For SHIP‐TREND‐0, we used its 5‐year follow‐up SHIP‐TREND‐1. For SHIP‐START, we used SHIP‐START‐1 as baseline (since no spirometry was available for SHIP‐START‐0), predicting incident airflow limitation at 5‐, 10 and 15‐years follow‐up (SHIP‐START‐2, SHIP‐START‐3 and SHIP‐START‐4).
**Figure S2:** Discriminative ability of Forced Expiratory Flow Measures for incident AL in KORA and SHIP. Receiver operating characteristic (ROC) curves were generated for small airway markers, such as FEF_25_, FEF_50_, FEF_75_ and FEF_25‐75_; in spirometrically healthy individuals (FEV/FVC > 10th percentile and > 0.7, FEV_1_ and FVC > 80% predicted and no history of chronic bronchitis). All values were adjusted for covariates using the GLI 2012 reference equations for FEF_25‐75_ and FEF_75_, and the LuftiBus ^31^ reference equations for FEF_25_ and FEF_50_. In addition, the unadjusted FEF50/FEF75 ratio was evaluated as a comparator of a non‐standardised small‐airways index. AUC values were indicated alongside respective confidence intervals. Baseline FEF_25‐75_ data were only available in KORA, restricting our analysis in SHIP to FEF_25_, FEF_50_, and FEF_75_ metrics. **A** ROC curves for the pooled KORA cohort (3‐year incident AL). **B** ROC curves for the pooled SHIP cohort (5‐year incident AL). AL: Airflow limitation.
**Figure S3:** Receiver Operating Characteristic (ROC) curve of the Random Forest classifier. The ROC curve illustrates the performance of the Random Forest Classifier evaluated on 25% of the entire dataset. The model achieved an Area Under the Curve (AUC) value of 0.87. The red dot indicates the optimal threshold based on the Youden's Index.
**Figure S4:** Confusion Matrix for the prediction of the validation data. The confusion matrix, based on the Youden's Index adjusted threshold for the Random Forest Classifier, indicates strong predictive performance of the validation with a sensitivity of 0.87 and a specificity of 0.81. The Matthews Correlation Coefficient (MCC) is 0.27, and the F1 score is 0.21.
**Figure S5:** Feature importances for prediction of incident AL using spirometry‐based at‐risk phenotypes and biomarkers. A random forest classifier was trained to predict incident AL across pooled data from KORA and SHIP cohorts (AUC = 0.87 on validation data). Input features included spirometry‐based variables (e.g., z‐FEV_1_, FVC, FEF_25‐75_, FEF_50_ and FEF_75_) and at‐risk phenotypes (early airflow limitation: EAL, FEF_25‐75_‐SAD: sad, FEF_75_‐SAD: FEF_75_‐SAD, FEF_50_‐SAD: preserved‐ratio impaired‐spirometry: PRISm). Interpretability of FEF_25‐75_ feature importance is limited due to its imputation for the SHIP cohort, where these measurements were not available. As expected, FEV_1_/FVC showed the highest predictive strength, followed by EAL and small airway markers, such as FEF75, FEF50. GLI2012 adjusted values are indicated by _z, values adjusted for LuftiBus reference equations are indicated by kuster_. AL: Airflow limitation.
**Figure S6:** Overlap of Small airway disease (SAD) definitions based on FEF_25‐75_, FEF_75_ and FEF_50_ in KORA and SHIP. Three definitions for SAD were derived using spirometric measures: FEF_25‐75_‐SAD, FEF_50_‐SAD and FEF_75_‐SAD. Specifically, these definitions classified individuals as having SAD if FEF_25‐75_, FEF_50_, or FEF_75_ were a predefined defined threshold, while other lung function parameters were within normal range (FEV_1_/FVC > 0.7 and > 10th percentile, FEV_1_ and FVC > 80% predicted). Before analysis, FEF_25‐75_ and FEF_75_ were standardised to z‐scores using GLI2012 reference equations^30^, while FEF_50_ was expressed in % predicted using LuftiBus equations^31^. Thresholds for FEF_50_ and FEF75 were optimised using Youden's index, whereas the FEF_25‐75_ threshold was based on a previous analysis by Kwon and colleagues^13^. (A) Overlap of SAD definitions in the pooled KORA dataset at baseline (*n* = 1973). (B) Overlap of SAD definitions in the pooled SHIP dataset at baseline (*n* = 4150). FEF_25‐75_ measurements were not available in SHIP‐START or SHIP‐Trend at baseline.

## Data Availability

For Ship, restrictions are imposed on the availability of data generated or analysed during this study to ensure the preservation of patient confidentiality or due to the utilisation of data under licence. The application for data can be made following a standardised procedure: http://www2.medizin.uni‐greifswald.de/cm/fv/ship/daten‐beantragen/. For Kora, data can be requested for research projects via the KORA.PASST use and access hub (https://helmholtz‐muenchen.managed‐otrs.com/external). The KORA Board is responsible for review and approval of the requests. The rights of study participants, adherence to Good Scientific Practice, and the goals of the KORA study guide the decision. The European General Data Protection Regulation (GDPR) applies to all applicants.

## References

[1] “Chronic Obstructive Pulmonary Disease (COPD)”, accessed May 28, 2025, https://www.who.int/news‐room/fact‐sheets/detail/chronic‐obstructive‐pulmonary‐disease‐(copd).

[2] M. K. Han , A. Agusti , B. R. Celli , et al., “From GOLD 0 to Pre‐COPD,” American Journal of Respiratory and Critical Care Medicine 203, no. 4 (2021): 414–423, 10.1164/rccm.202008-3328PP. PMC7885837 33211970

[3] S. Ananth and J. R. Hurst , “ERJ Advances: State of the Art in Definitions and Diagnosis of COPD,” European Respiratory Journal 61, no. 4 (2023): 2202318, 10.1183/13993003.02318-2022. 36796835

[4] E. Doña , R. Reinoso‐Arija , L. Carrasco‐Hernandez , A. Doménech , A. Dorado , and J. L. Lopez‐Campos , “Exploring Current Concepts and Challenges in the Identification and Management of Early‐Stage COPD,” Journal of Clinical Medicine 12, no. 16 (2023): 5293, 10.3390/jcm12165293. PMC10455125 37629335

[5] D. M. Mannino , E. Diaz‐Guzman , and S. Buist , “Pre‐ and Post‐Bronchodilator Lung Function as Predictors of Mortality in the Lung Health Study,” Respiratory Research 12, no. 1 (2011): 136, 10.1186/1465-9921-12-136. PMC3202237 21991942

[6] E. S. Wan , P. J. Castaldi , M. H. Cho , et al., “Epidemiology, Genetics, and Subtyping of Preserved Ratio Impaired Spirometry (PRISm) in COPDGene,” Respiratory Research 15, no. 1 (2014): 89, 10.1186/s12931-014-0089-y. PMC4256936 25096860

[7] S. R. A. Wijnant , E. De Roos , M. Kavousi , et al., “Trajectory and Mortality of Preserved Ratio Impaired Spirometry: The Rotterdam Study,” European Respiratory Journal 55, no. 1 (2020): 1901217, 10.1183/13993003.01217-2019. 31601717

[8] C. J. Cadham , H. Oh , M. K. Han , et al., “The Prevalence and Mortality Risks of PRISm and COPD in the United States From NHANES 2007–2012,” Respiratory Research 25, no. 1 (2024): 208, 10.1186/s12931-024-02841-y. PMC11096119 38750492

[9] Y. Çolak , P. Lange , J. Vestbo , B. G. Nordestgaard , and S. Afzal , “Susceptible Young Adults and Development of Chronic Obstructive Pulmonary Disease Later in Life,” American Journal of Respiratory and Critical Care Medicine 210, no. 5 (2024): 607–617, 10.1164/rccm.202308-1452OC. 38364200

[10] E. Turkeshi , B. Vaes , E. Andreeva , et al., “Airflow Limitation by the Global Lungs Initiative Equations in a Cohort of Very Old Adults,” European Respiratory Journal 46, no. 1 (2015): 123–132, 10.1183/09031936.00217214. 25882799

[11] Y. E. Odeyemi , O. Lewis , J. Ngwa , K. Dodd , R. F. Gillum , and A. Mehari , “Does Low FEV1 in Addition to Fixed Ratio and/or Lower Limit of Normal of FEV1/FVC Improve Prediction of Mortality in COPD? The NHANES‐III‐Linked‐Mortality Cohort,” Journal of the National Medical Association 111, no. 1 (2019): 94–100, 10.1016/j.jnma.2018.06.006. PMC6349529 30064691

[12] D. J. Tan , C. J. Lodge , E. H. Walters , et al., “Can We Use Lung Function Thresholds and Respiratory Symptoms to Identify Pre‐Chronic Obstructive Pulmonary Disease? A Prospective, Population‐Based Cohort Study,” American Journal of Respiratory and Critical Care Medicine 209, no. 12 (2024): 1431–1440, 10.1164/rccm.202212-2330OC. 38236192

[13] D. S. Kwon , Y. J. Choi , T. H. Kim , et al., “FEF25‐75% Values in Patients With Normal Lung Function Can Predict the Development of Chronic Obstructive Pulmonary Disease,” International Journal of Chronic Obstructive Pulmonary Disease 15 (2020): 2913–2921, 10.2147/COPD.S261732. PMC7669499 33209020

[14] N. Y. Alobaidi , M. Almeshari , J. Stockley , R. A. Stockley , and E. Sapey , “Small Airway Function Measured Using Forced Expiratory Flow Between 25% and 75% of Vital Capacity and Its Relationship to Airflow Limitation in Symptomatic Ever‐Smokers: A Cross‐Sectional Study,” BMJ Open Respiratory Research 9, no. 1 (2022): e001385, 10.1136/bmjresp-2022-001385. PMC9540854 36202407

[15] J. P. Allinson , “Chronic Bronchitis Revisited,” in COPD in the 21st Century, ed. J. A. Wedzicha , J. P. Allinson , and P. M. A. Calverley (European Respiratory Society, 2024).

[16] For the Canadian Cohort of Obstructive Lung Disease (CanCOLD) Study and the Canadian Respiratory Research Network , K. M. Johnson , W. C. Tan , J. Bourbeau , D. D. Sin , and M. Sadatsafavi , “The Diagnostic Performance of Patient Symptoms in Screening for COPD,” Respiratory Research 19, no. 1 (2018): 147, 10.1186/s12931-018-0853-5. PMC6090694 30075717

[17] A. Kaplan and M. Thomas , “Screening for COPD: The Gap Between Logic and Evidence,” European Respiratory Review 26, no. 143 (2017): 160113, 10.1183/16000617.0113-2016. PMC9489098 28298389

[18] Global Initiative for Chronic Obstructive Lung Disease ‐ GOLD , “GOLD Report”, accessed April 3, 2025 (2025), https://goldcopd.org/2025‐gold‐report/.

[19] R. Holle , M. Happich , H. Löwel , and H. Wichmann , “KORA ‐ A Research Platform for Population Based Health Research,” Das Gesundheitswesen 67, no. S 01 (2005): 19–25, 10.1055/s-2005-858235. 16032513

[20] A. Peters , A. Döring , K.‐H. Ladwig , et al., “Multimorbidität und erfolgreiches Altern: Ein Blick auf die Bevölkerung im Rahmen der KORA‐Age‐Studie,” Zeitschrift für Gerontologie und Geriatrie 44, no. S2 (2011): 41–54, 10.1007/s00391-011-0245-7. 22270973

[21] H. Völzke , “Study of Health in Pomerania (SHIP): Konzept, Kohortendesign und ausgewählte Ergebnisse,” Bundesgesundheitsblatt, Gesundheitsforschung, Gesundheitsschutz 55, no. 6–7 (2012): 790–794, 10.1007/s00103-012-1483-6. 22736157

[22] D. D. Sjoberg , K. Whiting , M. Curry , J. A. Lavery , and J. Larmarange , “Reproducible Summary Tables With the gtsummary Package,” R Journal 13, no. 1 (2021): 570, 10.32614/RJ-2021-053.

[23] N. V. Chawla , K. W. Bowyer , L. O. Hall , and W. P. Kegelmeyer , “SMOTE: Synthetic Minority Over‐Sampling Technique,” Journal of Artificial Intelligence Research 16 (2002): 321–357, 10.1613/jair.953.

[24] X. Robin , N. Turck , A. Hainard , et al., “pROC: Display and Analyze ROC Curves” (2010) 1.18.5.

[25] W. Viechtbauer , “Conducting Meta‐Analyses in *R* With the **metafor** Package,” Journal of Statistical Software 36, no. 3 (2024), 10.18637/jss.v036.i03.

[26] R. Martin and H. Pott , OnlineArts/PySpiro: v.0.2.0.

[27] P. H. Quanjer , S. Stanojevic , T. J. Cole , et al., “Multi‐Ethnic Reference Values for Spirometry for the 3–95 Year Age Range: The Global Lung Function 2012 Equations,” European Respiratory Journal 40, no. 6 (2012): 1324–1343, 10.1183/09031936.00080312. PMC3786581 22743675

[28] S. P. Kuster , D. Kuster , C. Schindler , et al., “Reference Equations for Lung Function Screening of Healthy Never‐Smoking Adults Aged 18–80 Years,” European Respiratory Journal 31, no. 4 (2008): 860–868, 10.1183/09031936.00091407. 18057057

[29] X. Robin , N. Turck , A. Hainard , et al., “pROC: An Open‐Source Package for R and S+ to Analyze and Compare ROC Curves,” BMC Bioinformatics 12, no. 1 (2011): 77, 10.1186/1471-2105-12-77. PMC3068975 21414208

[30] Global Initiative for Chronic Obstructive Lung Disease – GOLD , “GOLD Report”, accessed May 29, 2025 (2024), https://goldcopd.org/2024‐gold‐report/.

[31] B. L. Graham , I. Steenbruggen , M. R. Miller , et al., “Standardization of Spirometry 2019 Update. An Official American Thoracic Society and European Respiratory Society Technical Statement,” American Journal of Respiratory and Critical Care Medicine 200, no. 8 (2019): e70–e88, 10.1164/rccm.201908-1590ST. PMC6794117 31613151

[32] B. Knox‐Brown , O. Mulhern , J. Feary , and A. F. S. Amaral , “Spirometry Parameters Used to Define Small Airways Obstruction in Population‐Based Studies: Systematic Review,” Respiratory Research 23, no. 1 (2022): 67, 10.1186/s12931-022-01990-2. PMC8939095 35313875

[33] M. Llordés , A. Jaen , E. Zurdo , M. Roca Vilalta , I. Vazquez , and P. Almagro , “Fixed Ratio Versus Lower Limit of Normality for Diagnosing COPD in Primary Care: Long‐Term Follow‐Up of EGARPOC Study,” International Journal of Chronic Obstructive Pulmonary Disease 15 (2020): 1403–1413, 10.2147/COPD.S250720. PMC7308128 32606649

[34] A. Gogali and K. Kostikas , “Latent COPD: A Proposed New Term in the Disease Nomenclature,” European Respiratory Journal 61, no. 5 (2023): 2300535, 10.1183/13993003.00535-2023. 37208037

[35] B. R. Celli and A. Agustí , “COPD: Time to Improve Its Taxonomy?,” ERJ Open Research 4, no. 1 (2018): 00132‐02017, 10.1183/23120541.00132-2017. PMC5912933 29707563

[36] P. H. Quanjer , D. J. Weiner , J. J. Pretto , D. J. Brazzale , and P. W. Boros , “Measurement of FEF_25–75%_ and FEF_75%_ Does Not Contribute to Clinical Decision Making,” European Respiratory Journal 43, no. 4 (2014): 1051–1058, 10.1183/09031936.00128113. 24072211

[37] J. Fan , L. Fang , S. Cong , et al., “Potential Pre‐COPD Indicators in Association With COPD Development and COPD Prediction Models in Chinese: A Prospective Cohort Study,” Lancet Regional Health – Western Pacific 44 (2024): 100984, 10.1016/j.lanwpc.2023.100984. PMC10770747 38186582

