# Supplementary material for: Three Spirometry‐Based At‐Risk Phenotypes Predict Incident Airflow Limitation: A Population‐Based Study
Source: Respirology. 2026 Aug 11;31(11):1104–16. doi: 10.1002/resp.70296 (PMC13728517; doi:10.1002/resp.70296)
Supplement: Supplementary file 1 [file RESP-31-1104-s001.docx]

**SUPPLEMENT:**

# **Tables**

**Supp. Table 1: Characteristics of the pooled KORA and SHIP datasets.** Baseline and follow-up data for all 6123 patients included in the study were given. Datasets were generated from four German population-based cohorts: KORA-AGE (n = 841), KORA-F4 (n = 1,132), SHIP-START (n = 1,722, with SHIP-START-1 as baseline, as no spirometry was performed in SHIP-START-0), and SHIP-TREND (n = 2,428). All given spirometry values were sampled pre-bronchodilator. FEV_1_, FVC, FEV_1_/FVC, FEF75 and FEF25-75 were adjusted using the GLI2012 reference equations. FEF50 was adjusted to percent predicted using the LuftiBus reference equations^31^. Patients with baseline Asthma, COPD or chronic lung conditions other than chronic bronchitis were excluded. Follow-up data were available at three years for KORA-AGE, five years for SHIP-TREND, and three and six years for KORA-F4. FU: Follow-up. FEF_25-75_ data were not available for SHIP-START or SHIP-TREND at baseline. Smoking pack years were not available for baseline SHIP-START. AL: Airflow limitation.

**Supp. Table 2: Prevalences of at-risk phenotypes in the pooled exploration (KORA) and validation (SHIP) datasets.** Three frameworks of at-risk phenotypes (early airflow limitation, EAL, preserved-ratio impaired-spirometry, PRISm) were applied to KORA and SHIP, each varying by the used small airway dysfunction (SAD) phenotype. In total, three definitions for small airway dysfunction (SAD) were explored: FEF_25-75_-SAD, FEF_50_-SAD and FEF_75_-SAD. Specifically, these definitions classified individuals as having SAD if FEF_25-75_, FEF_50_, or FEF_75_ were below the Youden-optimised threshold, derived in the KORA dataset (in absence of EAL or PRISm: FEV1/FVC > 0.7 and > 10th percentile, FEV1 and FVC > 80 % predicted). For FEF_25-75_ and FEF_75_, thresholds were applied to the respective^30^, while the FEF_50_-threshold was applied to the percent predicted ratio (no z-scores available) from the LuftiBus equations^31^. FEF_25-75_ were unavailable for baseline SHIP and are only reported for KORA. Patients with available data for FEV1 and FVC but not for FEF metrics were classified as ‘Other’. Additionally, 144 individuals from the KORA dataset were older than 80 years, exceeding the valid range for the Luftibus equations, and were classified as ‘Other’.

**Supp. Table 3: Prevalences of at-risk phenotypes in patients younger and older than 50 years in KORA.** Baseline prevalence of at-risk phenotypes was compared between patients aged up to 50 years, and patients older than 50, using Pearson’s chi2 test. Post-hoc tests to compare individual subgroups were performed using Fisher’s exact test.

**Supp. Table 4: Prevalences of at-risk phenotypes in patients younger and older than 50 years in SHIP.** Baseline prevalence of at-risk phenotypes was compared between patients aged up to 50 years, and patients older than 50, using Pearson’s chi2 test. Post-hoc tests to compare individual subgroups were performed using Fisher’s exact test.

**Supp. Table 5: Association of TriSpi-FEF_75_ phenotypes with 3-/5-year incident AL in the pooled KORA and SHIP cohorts** The Table reports cases with and without incident ALover 3- and 5- year follow-up, stratified by baseline at-risk phenotypes in the pooled KORA and SHIP cohorts. Due to the low event count, we used Firth’s penalised logistic regression and limited the number of covariates to age, smoking status, sex, and the displayed at-risk phenotypes, to receive an acceptable Event/Covariate ratio. Covariates, Covariate/Event ratios, Odds ratios (OR), 95% confidence intervals (CI) and p-values are reported for all models. Regression Models were further adjusted for smoking pack years, which was not available for baseline SHIP-START, and thus not included in the pooled SHIP model. Patients classified as ‘Other’ and were excluded from the regression model to further shrink the number of covariates. AL: Airflow limitation.

**Supp. Table 6: Association of TriSpi-FEF_50_ phenotypes with 3-/5-year incident ALin the pooled KORA and SHIP cohorts** The Table reports cases with and without incident AL over 3- and 5- year follow-up, stratified by baseline at-risk phenotypes in the pooled KORA and SHIP cohorts. Due to the low event count, we used Firth’s penalised logistic regression and limited the number of covariates to age, smoking status, sex, and the displayed at-risk phenotypes, to receive an acceptable Event/Covariate ratio. Covariates, Covariate/Event ratios, Odds ratios (OR), 95% confidence intervals (CI) and p-values are reported for all models. Regression Models were further adjusted for smoking pack years, which was not available for baseline SHIP-START, and thus not included in the pooled SHIP model. Patients classified as ‘Other’ and were excluded from the regression model to further shrink the number of covariates. AL: Airflow limitation.

**Supp. Table 7: Both Discordant Airflow Limitation and Early Airflow Limitation with exceeded fixed-ratio criterion predict incident AL** As part of our sensitivity analyses, we split the EAL group into patients with discordant airflow limitation (FEV1/FVC < 0.7 but > 5th percentile (LLN)) and those with FEV1/FVC > 0.7 but < 10^th^ percentile. Associations with incident AL over 3- and 5- year follow-up were investigated using Firth’s penalised logistic regression. Covariates, Covariate/Event ratios, Odds ratios (OR), 95% confidence intervals (CI) and p-values are reported for all models. Regression Models were further adjusted for smoking pack years, which was not available for baseline SHIP-START, and thus not included in the pooled SHIP model. Patients classified as ‘Other’ and were excluded from the regression model to further shrink the number of covariates. AL: Airflow limitation.

**Supp. Table 8: Association of TriSpi-FEF_75_ phenotypes with 6-/10-/15-year incident AL in the pooled KORA and SHIP cohorts** The Table reports cases with and without incident AL over 6-, 10- and 15-year follow-up, stratified by baseline at-risk phenotypes in the pooled KORA and SHIP cohorts. Due to the low event count, we used Firth’s penalised logistic regression and limited the number of covariates to age, smoking status, sex, and the displayed at-risk phenotypes, to receive an acceptable Event/Covariate ratio. Covariates, Covariate/Event ratios, Odds ratios (OR), 95% confidence intervals (CI) and p-values are reported for all models. Regression Models were further adjusted for smoking pack years, which was not available for baseline SHIP-START, and thus not included in the pooled SHIP model. Patients classified as ‘Other’ and were excluded from the regression model to further shrink the number of covariates. AL: Airflow limitation.

**Supp. Table 9: Association of TriSpi-FEF_50_ phenotypes with 6-/10-/15-year incident AL in the pooled KORA and SHIP cohorts** The Table reports cases with and without incident AL over 6-, 10- and 15-year follow-up, stratified by baseline at-risk phenotypes in the pooled KORA and SHIP cohorts. Due to the low event count, we used Firth’s penalised logistic regression and limited the number of covariates to age, smoking status, sex, and the displayed at-risk phenotypes, to receive an acceptable Event/Covariate ratio. Covariates, Covariate/Event ratios, Odds ratios (OR), 95% confidence intervals (CI) and p-values are reported for all models. Regression Models were further adjusted for smoking pack years, which was not available for baseline SHIP-START, and thus not included in the pooled SHIP model. Patients classified as ‘Other’ and were excluded from the regression model to further shrink the number of covariates. AL: Airflow limitation.

**Supp. Table 10: Association of TriSpi-FEF_25-75_ phenotypes with 3-/6-year incident AL in KORA** The Table reports cases with and without incident AL over 3- and 6- year follow-up, stratified by baseline at-risk phenotypes in the KORA cohort. Two models were generated, one for the pooled KORA cohort (3-year FU), and one for the KORA-F4 cohort, which had a second follow up (6-year FU). We used Firth’s penalised logistic regression and limited the number of covariates to age, smoking status, sex, and the displayed at-risk phenotypes, to receive an acceptable Event/Covariate ratio. Covariates, Covariate/Event ratios, Odds ratios (OR), 95% confidence intervals (CI) and p-values are reported for all models.Patients classified as ‘Other’ and were excluded from the regression model to further shrink the number of covariates. AL: Airflow limitation.

**Supp. Table 11**: **Evaluating Variants of *TriSpi* for prediction of incident AL at longer follow-ups** We applied different variants of the TriSpi framework to the subsets of the KORA and SHIP cohorts with longer follow-up to evaluate their utility for prediction of incident ALat longer follow-ups. TriSpi is a set of three mutually exclusive definitions for spirometric at-risk phenotypes, encompassing early airflow limitation (EAL), preserved-ratio impaired-spirometry (PRISm) and small airway dysfunction (SAD). We systematically varied the definition used for SAD, comparing their clinical utility, using positive and negative predictive values (PPV, NPV), relative risk (RR), absolute risk reduction (ARR) and relative risk reduction (RRR), as well as the number needed to screen (NNS) to identify one case of incident ALin the general population as well as the TriSpi^+^-population. NNS was calculated as (Prevalence(TriSpi)_GeneralPopulation_ * Incidence(AL)_TriSpi-Patients_)^-1^ for the general population and (Incidence(AL)_TriSpi-Patients_)^-1^ for the TriSpi population. AL: Airflow limitation.

**Supp. Table 12**: **Benchmarking combinations of two at-risk phenotypes against the all three at-risk phenotypes** We assessed whether using three at-risk phenotypes (early airflow limitation, preserved-ratio impaired-spirometry, and small airway dysfunction), provided benefit over using two at-risk phenotypes. Combinations of two at-risk phenotypes were applied to both the KORA (3-year follow-up, 1476 patients) and SHIP (5-year follow-up, 2475 patients) cohorts. Sensitivity, specificity, positive predictive value (ppv) and negative predictive value (npv), were calculated for each combination. early airflow limitation: EAL, preserved-ratio impaired-spirometry: PRISm, small airway dysfunction based on FEF50: FEF50-SAD, small airway dysfunction based on FEF75: FEF75-SAD.

# **Figures**

**Supp. Figure 1: Included Cohorts, patients and respective follow-up times.** The given figure represents the patients in the original datasets of the four cohorts (KORA-AGE, KORA-F4, SHIP-TREND and SHIP-1), the numbers in the curated datasets, and the number of participants in the respective follow-up visits. Patients with Asthma, COPD or chronic lung disease at baseline were excluded, their numbers indicated by the respective cohort. Loss to Follow-up was similarly represented (*pt*. : Patients). No mortality data for dissection of Loss to Follow-up were available for this analysis. In KORA-AGE and -F4 patients completed a 3-years follow-up, plus an additional 6-year follow-up in KORA-F4. Follow-ups in both SHIP studies generally adhered to a 5-year interval. For SHIP-TREND-0, we used its 5-year follow-up SHIP-TREND-1. For SHIP-START, we used SHIP-START-1 as baseline (since no spirometry was available for SHIP-START-0), predicting incident airflow limitation at 5-, 10 and 15-years follow-up (SHIP-START-2, SHIP-START-3 and SHIP-START-4).

**Supp. Figure 2: Discriminative ability of Forced Expiratory Flow Measures for incident AL in KORA and SHIP.** Receiver operating characteristic (ROC) curves were generated for small airway markers, such as FEF_25_, FEF_50_, FEF_75_ and FEF_25-75_; in spirometrically healthy individuals (FEV/FVC > 10th percentile and > 0.7, FEV_1_and FVC > 80% predicted and no history of chronic bronchitis). All values were adjusted for covariates using the GLI 2012 reference equations for FEF_25-75_ and FEF_75_, and the LuftiBus^[31](https://www.zotero.org/google-docs/?2ZCA4T)^ reference equations for FEF_25_ and FEF_50_. In addition, the unadjusted FEF50/FEF75 ratio was evaluated as a comparator of a non-standardised small-airways index. AUC values were indicated alongside respective confidence intervals. Baseline FEF_25-75_ data were only available in KORA, restricting our analysis in SHIP to FEF_25_, FEF_50_, and FEF_75_ metrics. **A** ROC curves for the pooled KORA cohort (3-year incident AL). **B** ROC curves for the pooled SHIP cohort (5-year incident AL). AL: Airflow limitation.

**Supp. Figure 3: Receiver Operating Characteristic (ROC) curve of the Random Forest classifier.** The ROC curve illustrates the performance of the Random Forest Classifier evaluated on 25% of the entire dataset. The model achieved an Area Under the Curve (AUC) value of 0.87. The red dot indicates the optimal threshold based on the Youden's Index.

**Supp. Figure 4: Confusion Matrix for the prediction of the validation data.** The confusion matrix, based on the Youden's Index adjusted threshold for the Random Forest Classifier, indicates strong predictive performance of the validation with a sensitivity of 0.87 and a specificity of 0.81. The Matthews Correlation Coefficient (MCC) is 0.27, and the F1 score is 0.21.

**Supp. Figure 5: Feature importances for prediction of incident AL using spirometry-based at-risk phenotypes and biomarkers.** A random forest classifier was trained to predict incident AL across pooled data from KORA and SHIP cohorts (AUC = 0.87 on validation data). Input features included spirometry-based variables (e.g. z-FEV_1_, FVC, FEF_25-75_, FEF_50_ and FEF_75_) and at-risk phenotypes (early airflow limitation: eal, FEF_25-75_-SAD: sad, FEF_75_-SAD: FEF_75_-SAD, FEF_50_-SAD: preserved-ratio impaired-spirometry: PRISm). Interpretability of FEF_25-75_ feature importance is limited due to its imputation for the SHIP cohort, where these measurements were not available. As expected, FEV_1_/FVC showed the highest predictive strength, followed by EAL and small airway markers, such as FEF75, FEF50. GLI2012 adjusted values are indicated by _z, values adjusted for LuftiBus reference equations are indicated by kuster_. AL: Airflow limitation.

**Supp.** **Figure 6: Overlap of Small airway disease (SAD) definitions based on FEF_25-75_, FEF_75_ and FEF_50_ in KORA and SHIP.** Three definitions for SAD were derived using spirometric measures: FEF_25-75_-SAD, FEF_50_-SAD and FEF_75_-SAD. Specifically, these definitions classified individuals as having SAD if FEF_25-75_, FEF_50_, or FEF_75_ were a predefined defined threshold, while other lung function parameters were within normal range (FEV1/FVC > 0.7 and > 10th percentile, FEV1 and FVC > 80 % predicted). Before analysis, FEF_25-75_ and FEF_75_ were standardised to z-scores using GLI2012 reference equations^30^, while FEF_50_ was expressed in % predicted using LuftiBus equations^31^. Thresholds for FEF_50_ and FEF75 were optimised using Youden’s index, whereas the FEF_25-75_ threshold was based on a previous analysis by Kwon and colleagues^13^. **A** Overlap of SAD definitions in the pooled KORA dataset at baseline (n = 1,973). **B** Overlap of SAD definitions in the pooled SHIP dataset at baseline (n = 4,150). FEF_25-75_ measurements were not available in SHIP-START or SHIP-Trend at baseline.

## **Supp. Table 1**

| **Characteristic** | **KORA-AGE**   N = 841^1^ | **KORA-F4**   N = 1,132^1^ | **SHIP-START**  N = 1,722^1^ | **SHIP-TREND**   N = 2,428^1^ |
| --- | --- | --- | --- | --- |
| Age [a] | 76 (70, 81) | 52 (47, 57) | 52 (41, 63) | 53 (41, 64) |
| BMI [kg/m2] | 28.0 (25.5, 31.0) | 26.5 (24.1, 30.1) | 27.3 (24.4, 30.7) | 27.6 (24.7, 30.7) |
| Sex |  |  |  |  |
| Male | 424 (50%) | 531 (47%) | 842 (49%) | 1,222 (50%) |
| Female | 417 (50%) | 601 (53%) | 880 (51%) | 1,206 (50%) |
| Smoking Status |  |  |  |  |
| Current Smoker | 31 (3.7%) | 249 (22%) | 406 (24%) | 526 (22%) |
| Former Smoker | 325 (39%) | 419 (37%) | 556 (32%) | 965 (40%) |
| Never Smoker | 485 (58%) | 464 (41%) | 759 (44%) | 935 (39%) |
| Smoking Pack Years | 0 (0, 9) | 1 (0, 19) | 0 (0, 0) | 1 (0, 16) |
| z-FEV1 [GLI2012] | 0.47 (-0.23, 1.23) | 0.40 (-0.28, 1.08) | 0.21 (-0.47, 0.83) | 0.16 (-0.54, 0.81) |
| z-FVC [GLI2012] | 0.49 (-0.17, 1.19) | 0.41 (-0.19, 1.02) | -0.32 (-0.92, 0.27) | 0.08 (-0.54, 0.69) |
| z-FEV1/FVC [GLI2012] | -0.13 (-0.61, 0.34) | -0.07 (-0.56, 0.41) | 0.88 (0.29, 1.48) | 0.06 (-0.46, 0.55) |
| z-FEF75 [GLI2012] | 0.63 (0.16, 1.09) | 0.39 (-0.10, 0.86) | 0.79 (0.25, 1.32) | 0.39 (-0.20, 0.93) |
| z-FEF25-75 [GLI2012] | 0.10 (-0.52, 0.72) | 0.05 (-0.49, 0.62) | - | - |
| FEF50 % predicted [Kuster 2008] | 114 (89, 152) | 110 (90, 139) | 116 (95, 143) | 111 (88, 137) |
| Incident AL at 3-/5-years Follow-up | 17 (3.0%) | 24 (2.6%) | 22 (2.1%) | 70 (4.9%) |
| Incident AL at 6-/10-years Follow-up | - | 44 (5.1%) | 65 (7.9%) | - |
| ^1^Median (Q1, Q3); n (%) | | | | |

## **Supp. Table 2**

| **Characteristic** | **KORA**   N = 1,973^1^ | **SHIP**   N = 4,150^1^ |
| --- | --- | --- |
| Status-FEF_25-75_ |  |  |
| Healthy | 1,519 (77%) | - |
| EAL | 214 (11%) | - |
| Other | 1 (<0.1%) | - |
| Chronic Bronchitis | 55 (2.8%) | - |
| PRISm | 85 (4.3%) | - |
| FEF_25-75_-SAD | 99 (5.0%) | - |
| Status-FEF_50_ |  |  |
| Healthy | 1,088 (55%) | 2,650 (64%) |
| EAL | 214 (11%) | 171 (4.1%) |
| FEF_50_-SAD | 407 (21%) | 901 (22%) |
| Other | 145 (7.3%) | 8 (0.2%) |
| Chronic Bronchitis | 34 (1.7%) | 81 (2.0%) |
| PRISm | 85 (4.3%) | 339 (8.2%) |
| Status-FEF_75_ |  |  |
| Healthy | 1,467 (74%) | 3,046 (73%) |
| EAL | 214 (11%) | 171 (4.1%) |
| FEF_75_-SAD | 155 (7.9%) | 496 (12%) |
| Other | 0 (0%) | 8 (0.2%) |
| Chronic Bronchitis | 51 (2.6%) | 88 (2.1%) |
| PRISm | 85 (4.3%) | 339 (8.2%) |
| ^1^n (%) | | |

## **Supp. Table 3**

| **Characteristic** | **Age ≤** **50**  N = 490^1^ | **Age > 50**  N = 1,482^1^ | **p-value**^2^ |
| --- | --- | --- | --- |
| Status-FEF_50_ |  |  | <0.001 |
| Healthy | 309 (63%) | 778 (52%) |  |
| EAL | 31 (6.3%) | 183 (12%) |  |
| FEF_50_-SAD | 124 (25%) | 283 (19%) |  |
| Other | 0 (0%) | 145 (9.8%) |  |
| Chronic Bronchitis | 8 (1.6%) | 26 (1.8%) |  |
| PRISm | 18 (3.7%) | 67 (4.5%) |  |
| Status-FEF_75_ |  |  | <0.001 |
| Healthy | 356 (73%) | 1,111 (75%) |  |
| EAL | 31 (6.3%) | 183 (12%) |  |
| FEF_75_-SAD | 75 (15%) | 80 (5.4%) |  |
| Chronic Bronchitis | 10 (2.0%) | 41 (2.8%) |  |
| PRISm | 18 (3.7%) | 67 (4.5%) |  |
| Status-FEF_25-75_ |  |  | <0.001 |
| Healthy | 393 (80%) | 1,126 (76%) |  |
| EAL | 31 (6.3%) | 183 (12%) |  |
| Chronic Bronchitis | 11 (2.2%) | 44 (3.0%) |  |
| PRISm | 18 (3.7%) | 67 (4.5%) |  |
| FEF_25-75_-SAD | 37 (7.6%) | 62 (4.2%) |  |
| ^1^n (%) | | | |
| ^2^Pearson's Chi-squared test | | | |

## **Supp. Table 4**

| **Characteristic** | **Age ≤** **50**  N = 1,851^1^ | **Age > 50**  N = 2,284^1^ | **p-value**^2^ |
| --- | --- | --- | --- |
| Status-FEF_50_ |  |  | <0.001 |
| Healthy | 1,213 (66%) | 1,436 (63%) |  |
| EAL | 84 (4.5%) | 87 (3.8%) |  |
| FEF_50_-SAD | 421 (23%) | 474 (21%) |  |
| Chronic Bronchitis | 27 (1.5%) | 54 (2.4%) |  |
| PRISm | 106 (5.7%) | 233 (10%) |  |
| Status-FEF_75_ |  |  | <0.001 |
| Healthy | 1,303 (70%) | 1,738 (76%) |  |
| EAL | 84 (4.5%) | 87 (3.8%) |  |
| FEF_75_-SAD | 335 (18%) | 161 (7.1%) |  |
| Chronic Bronchitis | 23 (1.2%) | 65 (2.8%) |  |
| PRISm | 106 (5.7%) | 233 (10%) |  |
| ^1^n (%) | | | |
| ^2^Pearson's Chi-squared test | | | |

## **Supp. Table 5**

| Cohort / Subgroup | Incident AL/n per cohort | Phenotype | Cases incident AL/n per Phenotype | Covariates | Event/Covariate ratio | Firth’s penalised regression model | | |
| --- | --- | --- | --- | --- | --- | --- | --- | --- |
|  |  |  |  |  |  | OR | 95% CI | p |
| Pooled KORA (3-year Follow-up) | 40 / 1,441 | Healthy | 5 / 1,086 | Age, Smoking Status, Sex, Smoking Pack Years | 5 | Control | | |
|  |  | EAL | 27 / 136 |  |  | 53.2 | 21.7 - 155.3 | < 0.0001 |
|  |  | Chronic Bronchitis | 0 / 36 |  |  | 2.57 | 0.02 - 23.6 | 0.6 |
|  |  | PRISm | 1 / 53 |  |  | 5.04 | 0.51 - 26.3 | 0.14 |
|  |  | FEF_75_-SAD | 7 / 130 |  |  | 9.21 | 2.93 - 30.7 | 0.0002 |
| Pooled SHIP (5-year Follow-up) | 92 / 2470 | Healthy | 22 / 1,887 | Age, Smoking Status, Sex | 13.1 | Control | | |
|  |  | EAL | 34 / 91 |  |  | 46.36 | 25.6 - 85.8 | < 0.0001 |
|  |  | Chronic Bronchitis | 2 / 45 |  |  | 3.99 | 0.8 - 13.03 | 0.087 |
|  |  | PRISm | 9 / 157 |  |  | 5.12 | 2.24 - 10.86 | 0.0003 |
|  |  | FEF_75_-SAD | 25 / 290 |  |  | 7.75 | 4.28 - 14.14 | < 0.0001 |

## **Supp. Table 6**

| Cohort / Subgroup | Incident AL/n per cohort | Phenotype | Cases incident AL/n per Phenotype | Covariates | Event/Covariate ratio | Firth’s penalised regression model | | |
| --- | --- | --- | --- | --- | --- | --- | --- | --- |
|  |  |  |  |  |  | OR | 95% CI | p |
| Pooled KORA (3-year Follow-up) | 40 / 1,369 | Healthy | 3 / 836 | Age, Smoking Status, Sex, Smoking Pack Years | 5 | Control | | |
|  |  | EAL | 27 / 136 |  |  | 75.81 | 26.3 - 249.8 | < 0.0001 |
|  |  | Chronic Bronchitis | 0 / 25 |  |  | 3.67 | 0.03 - 40.37 | 0.5 |
|  |  | PRISm | 1 / 53 |  |  | 6.78 | 0.64 - 43.12 | 0.097 |
|  |  | FEF_50_-SAD | 9 / 319 |  |  | 9.68 | 2.86 - 40.73 | 0.0002 |
| Pooled SHIP (5-year Follow-up) | 92 / 2475 | Healthy | 15 / 1,674 | Age, Smoking Status, Sex | 13.1 | Control | | |
|  |  | EAL | 34 / 91 |  |  | 64.4 | 33.41 - 129.3 | < 0.0001 |
|  |  | Chronic Bronchitis | 1 / 42 |  |  | 2.92 | 0.31 - 12.42 | 0.3 |
|  |  | PRISm | 157 |  |  | 6.99 | 2.94 | < 0.0001 |
|  |  | FEF_50_-SAD | 33 / 511 |  |  | 9.40 | 5.0 - 18.37 | < 0.0001 |

## **Supp. Table 7**

| Cohort / Subgroup | Incident AL/n per cohort | Phenotype | Cases incident AL/n per Phenotype | Covariates | Event/Covariate ratio | Firth’s penalised regression model | | |
| --- | --- | --- | --- | --- | --- | --- | --- | --- |
|  |  |  |  |  |  | OR | 95% CI | p |
| Pooled KORA (3-year Follow-up) | 40 / 1,369 | Healthy | 3 / 836 | Age, Smoking Status, Smoking Pack Years | 5 | Control | | |
|  |  | Discordant Airflow Limitation (EAL with FEV1/FVC < 0.7) | 20 / 111 |  |  | 62.95 | 19.62 - 236.86 | < 0.0001 |
|  |  | EAL (EAL with FEV1/FVC > 0.7) | 7 / 25 |  |  | 75.51 | 19.76 - 345.93 | < 0.0001 |
|  |  | Chronic Bronchitis | 0 / 25 |  |  | 3.91 | 0.03 - 42.67 | 0.5 |
|  |  | PRISm | 1 / 53 |  |  | 5.85 | 0.55 - 36.85 | 0.1 |
|  |  | FEF_50_-SAD | 9 / 319 |  |  | 6.98 | 2.19 - 28.15 | 0.0009 |
| Pooled SHIP (5-year Follow-up) | 92 / 2475 | Healthy | 15 / 1674 | Age, Smoking Status, Sex |  | Control | | |
|  |  | Discordant Airflow Limitation (EAL with FEV1/FVC < 0.7) | 16 / 36 |  |  | 81.07 | 34.48 - 195.07 | < 0.0001 |
|  |  | EAL (EAL with FEV1/FVC > 0.7) | 18 / 55 |  |  | 53.12 | 23.66 - 122.00 | < 0.0001 |
|  |  | Chronic Bronchitis | 1 / 42 |  |  | 2.96 | 0.32 - 12.56 | 0.3 |
|  |  | PRISm | 9 / 157 |  |  | 7.03 | 2.96 - 15.93 | < 0.0001 |
|  |  | FEF_50_-SAD | 33 / 511 |  |  | 9.22 | 4.91 - 18.04 | < 0.0001 |

## **Supp. Table 8**

| Cohort / Subgroup | Incident AL/n per cohort | Phenotype | Cases incident AL/n per Phenotype | Covariates | Event/Covariate ratio | Firth’s penalised regression model | | |
| --- | --- | --- | --- | --- | --- | --- | --- | --- |
|  |  |  |  |  |  | OR | 95% CI | p |
| KORA-F4 (6-year Follow-up) | 44/834 | Healthy | 8 / 640 | Age, Smoking Status, Sex, Smoking Pack Years | 5.5 | Control | | |
|  |  | EAL | 18 /41 |  |  | 61.98 | 25.06 - 166.1 | < 0.0001 |
|  |  | Chronic Bronchitis | 0 / 19 |  |  | 1.63 | 0.01 - 14.40 | 0.8 |
|  |  | PRISm | 3 / 32 |  |  | 6.96 | 1.61 - 24.65 | 0.0124 |
|  |  | FEF_75_-SAD | 15 / 102 |  |  | 10.41 | 4.32 - 26.58 | < 0.0001 |
| SHIP-START (10-year Follow-up) | 65 / 819 | Healthy | 36 / 678 | Age, Smoking Status, Sex | 9.3 | Control | | |
|  |  | EAL | 6 / 8 |  |  | 47.9 | 11.4 - 274.38 | < 0.0001 |
|  |  | Chronic Bronchitis | 1 / 11 |  |  | 1.95 | 0.20 - 9.02 | 0.5 |
|  |  | PRISm | 5 / 57 |  |  | 1.79 | 0.62 - 4.32 | 0.26 |
|  |  | FEF_75_-SAD | 17 / 65 |  |  | 5.90 | 2.93 - 11.66 | < 0.0001 |
| SHIP-START (15-year Follow-up)) | 27 / 645 | Healthy | 14 / 538 | Age, Smoking Status | 4.5 | Control | | |
|  |  | EAL | 1 / 4 |  |  | 15.82 | 1.17 - 135.16 | 0.0396 |
|  |  | Chronic Bronchitis | 0 / 7 |  |  | 1.39 | 0.01 - 14.02 | 0.8 |
|  |  | PRISm | 2 / 41 |  |  | 1.93 | 0.36 - 6.90 | 0.4 |
|  |  | FEF_75_-SAD | 10 / 55 |  |  | 6.44 | 2.51 - 16.22 | 0.0002 |

## **Supp. Table 9**

| Cohort / Subgroup | Incident AL/n per cohort | Phenotype | Cases incident AL/n per Phenotype | Covariates | Event/Covariate ratio | Firth’s penalised regression model | | |
| --- | --- | --- | --- | --- | --- | --- | --- | --- |
|  |  |  |  |  |  | OR | 95% CI | p |
| KORA-F4 (6-year Follow-up) | 44 / 857 | Healthy | 7 / 532 | Age, Smoking Status, Sex, Smoking Pack Years | 5.5 | Control | | |
|  |  | EAL | 18 / 41 |  |  | 93.04 | 32.2 - 282.1 | < 0.0001 |
|  |  | Chronic Bronchitis | 0 / 16 |  |  | 1.70 | 0.01 - 16.42 | 0.7 |
|  |  | PRISm | 3 / 32 |  |  | 7.97 | 1.76 - 30.38 | 0.0096 |
|  |  | FEF_50_-SAD | 16 / 213 |  |  | 12.08 | 4.65 - 34.20 | < 0.0001 |
| SHIP-START (10-year Follow-up) | 65 / 819 | Healthy | 11 / 587 | Age, Smoking Status, Sex | 9.3 | Control | | |
|  |  | EAL | 6 / 8 |  |  | 113.7 | 24.94 - 693 | < 0.0001 |
|  |  | Chronic Bronchitis | 0 / 11 |  |  | 1.61 | 0.01 - 14.54 | 0.8 |
|  |  | PRISm | 5 /57 |  |  | 5.32 | 1.70 - 14.85 | 0.0058 |
|  |  | FEF_50_-SAD | 43 / 156 |  |  | 21.25 | 10.44 - 46.71 | < 0.0001 |
| SHIP-START (15-year Follow-up)) | 27 / 645 | Healthy | 8 / 472 | Age, Smoking Status | 4.5 | Control | | |
|  |  | EAL | 1 / 4 |  |  | 22.9 | 1.62 - 211.52 | 0.0242 |
|  |  | Chronic Bronchitis | 0 / 8 |  |  | 1.69 | 0.01 - 17.40 | 0.8 |
|  |  | PRISm | 2 / 41 |  |  | 2.92 | 0.52 - 11.58 | 0.1957 |
|  |  | FEF_50_-SAD | 16 / 129 |  |  | 7.92 | 3.35 - 20.00 | < 0.0001 |

## **Supp. Table 10**

| Cohort / Subgroup | Incident AL/n per cohort | Phenotype | Cases incident AL/n per Phenotype | Covariates | Event/Covariate ratio | Firth’s penalised regression model | | |
| --- | --- | --- | --- | --- | --- | --- | --- | --- |
|  |  |  |  |  |  | OR | 95% CI | p |
| Pooled KORA (3-year Follow-up) | 40 / 1,440 | Healthy | 7 / 1,131 | Age, Smoking Status, Sex, Smoking Pack Years | 5 | Control | | |
|  |  | EAL | 27 / 136 |  |  | 41.53 | 18.26 - 105.83 | < 0.0001 |
|  |  | Chronic Bronchitis | 0 / 40 |  |  | 1.72 | 0.01 - 14.76 | 0.7 |
|  |  | PRISm | 1 / 53 |  |  | 3.80 | 0.39 - 18.17 | 0.2 |
|  |  | FEF_25-75_-SAD | 5 / 80 |  |  | 9.04 | 2.71 - 23.86 | 0.0007 |
| KORA-F4 (6-year Follow-up) | 44 / 834 | Healthy | 11 / 683 | Age, Smoking Status, Sex | 5.5 | Control | | |
|  |  | EAL | 18 / 41 |  |  | 53.30 | 22.42 - 134.06 | < 0.0001 |
|  |  | Chronic Bronchitis | 0 / 21 |  |  | 1.15 | 0.01 - 9.85 | 0.9 |
|  |  | PRISm | 3 / 32 |  |  | 5.51 | 1.30 - 18.50 | 0.023 |
|  |  | FEF_25-75_-SAD | 12 / 57 |  |  | 15.79 | 6.39 - 39.59 | < 0.0001 |

## **Supp. Table 11**

| Cohort | SAD definition | Incidence of AL: n (%) | | PPV | NPV | RR | ARR (TriSpi^-^) | RRR (TriSpi^-^) | NNS  (General Population) | NNS  (TriSpi^+^) |
| --- | --- | --- | --- | --- | --- | --- | --- | --- | --- | --- |
|  |  | TriSpi^+^ | TriSpi^-^ |  |  |  |  |  |  |  |
| KORA-F4  (6-y FU) | FEF_75_ | 36 (20.57) | 8 (1.17) | 20.6 | 98.8 | 17.5 | -19.40 | -94.30 | 23.8 | 4.9 |
|  | FEF_50_ | 37 (12.80) | 7 (1.23) | 12.8 | 98.8 | 10.4 | -11.57 | -90.37 | 23.2 | 7.8 |
|  | FEF_50_  or FEF_75_ | 39 (11.89) | 5 (0.71) | 11.9 | 99.1 | 16.7 | -11.18 | -94.03 | 22.0 | 8.4 |
|  | FEF_50_  and FEF_75_ | 34 (25.00) | 10 (1.39) | 25.0 | 98.6 | 18.0 | -23.61 | 94.45 | 25.2 | 4.0 |
| SHIP-START  (10-y FU) | FEF_75_ | 28 (21.54) | 37 (5.37) | 21.5 | 94.6 | 4.0 | -16.17 | -75.07 | 29.2 | 4.6 |
|  | FEF_50_ | 54 (24.43) | 11 (1.84) | 24.4 | 98.2 | 13.3 | -22.59 | -92.47 | 15.2 | 4.1 |
|  | FEF_50_  or FEF_75_ | 55 (23.01) | 10 (1.41) | 23.0 | 98.3 | 16.3 | -21.60 | -93.88 | 14.9 | 4.3 |
|  | FEF_50_  and FEF_75_ | 27 (24.11) | 38 (5.37) | 24.1 | 94.6 | 4.5 | -18.73 | -77.70 | 30.3 | 4.1 |
| SHIP-START  (15-y FU) | FEF_75_ | 13 (13.00) | 14 (2.57) | 13.0 | 97.4 | 5.1 | -10.43 | 80.24 | 49.6 | 7.7 |
|  | FEF_50_ | 19 (11.52) | 8 (1.67) | 11.5 | 98.3 | 6.9 | -9.85 | 85.53 | 33.9 | 8.7 |
|  | FEF_50_  or FEF_75_ | 21 (11.60) | 6 (1.06) | 11.6 | 98.7 | 10.9 | -10.54 | -90.83 | 30.7 | 8.6 |
|  | FEF_50_  and FEF_75_ | 11 (13.10) | 16 (2.85) | 13.1 | 97.1 | 4.6 | -10.24 | 78.22 | 58.6 | 7.6 |

## **Supp. Table 12**

| Study | At-risk Phenotypes | n | events | sensitivity | specificity | ppv | npv |
| --- | --- | --- | --- | --- | --- | --- | --- |
| SHIP | EAL, PRISm, FEF50-SAD | 2475 | 92 | 82,6 | 69,4 | 9,1 | 99,1 |
|  | EAL, FEF50-SAD |  |  | 81,5 | 73,5 | 10,6 | 99 |
|  | PRISM, FEF50-SAD |  |  | 81,5 | 71,6 | 10 | 99 |
|  | EAL, PRISm, FEF75-SAD |  |  | 73,9 | 78,3 | 11,2 | 98,8 |
|  | EAL, FEF75-SAD |  |  | 70,7 | 83,2 | 14 | 98,7 |
|  | PRISm, FEF75-SAD |  |  | 70,7 | 81,2 | 12,7 | 98,6 |
|  | EAL, PRISm |  |  | 46,7 | 91,4 | 17,3 | 97,8 |
| KORA | EAL, PRISm, FEF50-SAD | 1476 | 41 | 92,7 | 65 | 6,9 | 99,7 |
|  | EAL, FEF50-SAD |  |  | 90,2 | 67,7 | 7,4 | 99,6 |
|  | EAL, PRISm, FEF75-SAD |  |  | 87,8 | 78,2 | 10,1 | 99,6 |
|  | EAL, FEF75-SAD |  |  | 85,4 | 81,7 | 11,7 | 99,5 |
|  | PRISM, FEF50-SAD |  |  | 78 | 69,1 | 6,7 | 99,1 |
|  | EAL, PRISm |  |  | 70,7 | 88,6 | 15,1 | 99,1 |
|  | PRISm, FEF75-SAD |  |  | 63,4 | 84 | 10,2 | 98,8 |

## **Supp. Fig 1**

## **Supp. Fig 2**

## **Supp. Fig 3**

## **Supp. Fig 4**

## **Supp. Fig 5**

## **Supp. Fig 6**
